# Reassessment of Morphological Diagnostic Characters and Species Boundaries Requires Taxonomical Changes for the Genus *Orthopyxis* L. Agassiz, 1862 (Campanulariidae, Hydrozoa) and Some Related Campanulariids

**DOI:** 10.1371/journal.pone.0117553

**Published:** 2015-02-27

**Authors:** Amanda F. Cunha, Gabriel N. Genzano, Antonio C. Marques

**Affiliations:** 1 Departamento de Zoologia, Instituto de Biociências, Universidade de São Paulo, São Paulo, Brazil; 2 Estación Costera Nágera, Dpto. Cs. Marinas, Instituto de Investigaciones Marinas y Costeras (IIMyC), CONICET, Universidad Nacional de Mar del Plata, Mar del Plata, Argentina; 3 Centro de Biologia Marinha, Universidade de São Paulo, São Sebastião, São Paulo, Brazil; Universidade Federal do Rio de Janeiro, BRAZIL

## Abstract

The genus *Orthopyxis* is widely known for its morphological variability, making species identification particularly difficult. A number of nominal species have been recorded in the southwestern Atlantic, although most of these records are doubtful. The goal of this study was to infer species boundaries in the genus *Orthopyxis* from the southwestern Atlantic using an integrative approach. Intergeneric limits were also tested using comparisons with specimens of the genus *Campanularia*. We performed DNA analyses using the mitochondrial genes 16S and COI and the nuclear ITS1 and ITS2 regions. *Orthopyxis* was monophyletic in maximum likelihood analyses using the combined dataset and in analyses with 16S alone. Four lineages of *Orthopyxis* were retrieved for all analyses, corresponding morphologically to the species *Orthopyxis sargassicola* (previously known in the area), *Orthopyxis crenata* (first recorded for the southwestern Atlantic), *Orthopyxis caliculata* (= *Orthopyxis minuta* Vannucci, 1949 and considered a synonym of *O. integra* by some authors), and *Orthopyxis mianzani* sp. nov. A re-evaluation of the traditional morphological diagnostic characters, guided by our molecular analyses, revealed that *O. integra* does not occur in the study area, and *O. caliculata* is the correct identification of one of the lineages occurring in this region, corroborating the validity of that species. *Orthopyxis mianzani* sp. nov. resembles *O. caliculata* with respect to gonothecae morphology and a smooth hydrothecae rim, although it shows significant differences for other characters, such as perisarc thickness, which has traditionally been thought to have wide intraspecific variation. The species *O. sargassicola* is morphologically similar to *O. crenata*, although they differ in gonothecae morphology, and these species can only be reliably identified when this structure is present.

## Introduction

Hydroids of the family Campanulariidae Johnston, 1836 (Hydrozoa, Cnidaria) are ubiquitous in marine benthic communities, and in the southwestern Atlantic, they are frequently recorded in ecological and faunal studies [1,2,3,4,5,6,7,8,9,10,11,12,13]. Formal taxonomical studies of this family are relatively rare and mainly address the evolution of the medusa [14,15,16,17] and the delimitation of genera and species [7,18,19,20,21,22,23,24,25]. There has been a clear discordance regarding the diagnostic morphological characters used in the taxonomy of this group [19,26,27,28,29,30,31], mostly because the majority of these species have simple and similar morphologies that can be quite variable cf. [19]. In addition, the phylogenetic position of the family Campanulariidae among the Leptothecata cf. [32,33,34] is currently under dispute [17,35,36].

The genus *Orthopyxis* L. Agassiz, 1862 clearly illustrates the difficulties associated with taxa delimitation in the family. Many uncertainties exist concerning the validity of this genus e.g., [19,26,28,29,37,38], and it has been synonymized multiple times with the genus *Campanularia* Lamarck, 1816 based on their morphological similarities. In addition, species traditionally assigned to the genus *Orthopyxis* have very similar morphologies and few diagnostic characters, making delimitation difficult, particularly when only trophosomal characters are considered or available cf. [27,39]. Altogether, these practical issues—particularly the uncertain validity of the genus e.g., [19] (p.60) and many of its species e.g., [14,19]—demand different taxonomic approaches to reassess and establish species boundaries within *Orthopyxis*.

In the southwestern Atlantic, five species of the genus *Orthopyxis* have been recorded along the coast of Brazil by Vannucci-Mendes [40] and Vannucci [41,42], which were later re-identified as two species: *Orthopyxis integra* (Macgillivray, 1842) and *Orthopyxis sargassicola* (Nutting, 1915) [1,13,31] (Table 1). Vannucci-Mendes [40] and Vannucci [42] also recorded two species of *Campanularia* along the southeastern coast of Brazil, although both records are now considered dubious [8]. Unfortunately, a formal revision of these records is not possible, as most of the materials described by Vannucci have been lost [1]. Along the Argentinean coast, Blanco [43,44,45] recorded several species of *Campanularia* and *Orthopyxis*, some of which she subsequently re-identified as *Campanularia subantarctica* Millard, 1971 [46], which is currently considered to be a synonym of *Campanularia lennoxensis* Jäderholm, 1903 [47] (Table 1). Other records of *Campanularia* and *Orthopyxis* for the southwestern Atlantic are listed in Table 1. Most of them are considered dubious, requiring a revision of species records in this region.

**Table 1:** Records of species of *Orthopyxis* and *Campanularia* from the southwestern Atlantic, including their reidentifications, according to the literature.

Record	Author of the record	Locality of the record	Reidentification	Author of the reindentification
*Campanularia agas* Cornelius, 1982	[3,4,6,9,131, 132]	Uruguay and Argentina	-	-
*Campanularia caliculata* Hincks, 1853	[133]	Strait of Magellan	*Orthopyxis caliculata* (Hincks, 1853)	[43]
			*Orthopyxis integra* (Macgillivray, 1842)	[150]
			? *Orthopyxis crenata* (Hartlaub, 1901)	[47]
*Campanularia clytioides* (Lamouroux, 1824)	[133]	Strait of Magellan	-	-
*Campanularia compressa* Clark, 1876	[134]	Tierra del Fuego and Falkland Islands	*Campanularia integra* Macgillivray, 1842	[46,130]
*Campanularia (Orthopyxis) everta* Clark, 1876	[45]	Tierra del Fuego, Argentina	*Campanularia subantarctica* Millard, 1971	[46]
			*Orthopyxis mollis* (Stechow, 1919)	[97,150]
			*Campanularia lennoxensis* Jäderholm, 1903	[47]
			*Orthopyxis hartlaubi* El Beshbeeshy, 2011	[138]
			*Campanularia hartlaubi* (El Beshbeeshy, 2011)	[56]
	[135]	Between Falkland Islands and Tierra del Fuego; Strait of Magellan	*Campanularia subantarctica* Millard, 1971	[46]
			*Orthopyxis mollis* (Stechow, 1919)	[97]
			*Orthopyxis hartlaubi* El Beshbeeshy, 2011	[138]
			*Campanularia lennoxensis* Jäderholm, 1903	[56]
*Campanularia everta* Clark, 1876	[130]	Argentina	-	-
*Campanularia hesperia* Torrey, 1904	[8,40,89,136]	Santo Amaro Island, São Paulo, Brazil	? *Campanularia hesperia* Torrey, 1904	[1,8]
*Campanularia hincksii* Alder, 1856	[10,12,53]	Rio de Janeiro and Bahia, Brazil	-	-
	[3,6,9,57,58, 130, 137,138]	Argentina; Mar del Plata, Buenos Aires, Argentina	-	-
*Campanularia hincksii grandis* Billard, 1906	[139]	Quequén, Buenos Aires, Argentina	*Campanularia hincksii* Alder, 1856	[46,57,138]
*Campanularia hicksoni* Totton, 1930	[137]	Tierra del Fuego, Argentina	? *Campanularia hicksoni* Totton, 1930	[151]
	[138,140]	Tierra del Fuego and Beagle Channel	-	-
*Campanularia integra* Macgillivray, 1842	[43,46,140]	Punta Peñas, Santa Cruz, Argentina and Beagle Channel	-	-
*Campanularia* (*Campanularia*) *laevis* Hartlaub, 1905	[135]	Strait of Magellan, Argentina	*Campanularia agas* Cornelius, 1982	[19,130]
*Campanularia laevis* Hartlaub, 1905	[42]	Cabo Frio, Rio de Janeiro, Brazil	? *Campanularia agas* Cornelius, 1982	[1,8]
	[137,138]	Buenos Aires, Argentina	*Campanularia agas* Cornelius, 1982	[150]
*Campanularia lennoxensis* Jäderholm, 1903	[141,142]	Rio de Janeiro, Brazil	*Orthopyxis crenata* (Hartlaub, 1901)	[42]
			? *Orthopyxis sargassicola* (Nutting, 1915)	[1]
*Campanularia longitheca* Stechow, 1924	[143]	Falkland Islands; Strait of Magellan	? *Campanularia (Orthopyxis) everta* Clark, 1876	[45]
*Campanularia* (*Orthopyxis*) *norvegiae* Broch, 1948	[46,144]	South Georgia Islands	-	-
*Campanularia* sp.	[145]	Bahía San Sebastián, Tierra del Fuego, Argentina	-	-
*Campanularia subantarctica* Millard, 1971	[6,46,57,58, 88,129,140]	Mar del Plata, Golfo San Matías, Golfo San Jorge, Tierra del Fuego, and Isla de los Estados, Argentina; Canal Beagle	-	-
*Campanularia volubilis* (Linnaeus, 1758) var. *antarctica* Ritchie, 1913	[43,130]	Punta Peñas, San Julián, Argentina	? *Campanularia antarctica* Ritchie, 1913	[151]
*Campanularia tincta* Hincks, 1861	[133]	Falkland Islands	?*Campanularia tincta* Hincks, 1861	[28]
			*Campanularia longitheca* Stechow, 1924	[143]
			*Campanularia subantarctica* Millard, 1971	[46]
			*Orthopyxis mollis* (Stechow, 1919)	[97,150]
			*Orthopyxis hartlaubi* El Beshbeeshy, 2011	[138]
			*Campanularia hartlaubi* (El Beshbeeshy, 2011)	[56]
	[134]	Falkland Islands	?*Campanularia tincta* Hincks, 1861	[28]
			*Campanularia longitheca* Stechow, 1924	[143]
			*Campanularia subantarctica* Millard, 1971	[46]
	[146]	Falkland Islands	*Campanularia longitheca* Stechow, 1924	[143]
			*Campanularia subantarctica* Millard, 1971	[46]
	[147]	Tierra del Fuego, Argentina	*Campanularia longitheca* Stechow, 1924	[143]
			*Campanularia subantarctica* Millard, 1971	[46]
			*Campanularia hartlaubi* (El Beshbeeshy, 2011)	[56]
	[43]	Punta Peñas, Santa Cruz, Argentina	*Campanularia* (*Orthopyxis*) *everta* Clark, 1876	[45]
			*Campanularia subantarctica* Millard, 1971	[46]
*Campanularia tincta* Hincks, 1861 var. *eurycalyx* Hartlaub, 1905	[133]	Falkland Islands	*Campanularia eurycalyx* Stechow, 1924	[130,143]
			*Campanularia subantarctica* Millard, 1971	[46]
			*Orthopyxis mollis* (Stechow, 1919)	[150]
			? *Campanularia lennoxensis* Jäderholm, 1903	[47]
*Eucopella crenata* Hartlaub, 1901	[133]	Tierra del Fuego, Argentina	*Orthopyxis lennoxensis* (Jäderholm, 1903)	[40,130]
			? *Campanularia (Orthopyxis) everta* Clark, 1876	[45,135]
			*Orthopyxis mollis* (Stechow, 1919)	[150]
			*Campanularia lennoxensis* Jäderholm, 1903	[47]
*Orthopyxis billardi* Vannucci, 1954	[42]	São João da Barra, Rio de Janeiro, Brazil	*Orthopyxis sargassicola* (Nutting, 1915)	[31](?), [1,8,13]
*Orthopyxis caliculata* (Hincks, 1853)	[43]	Puerto Madryn, Argentina	*Campanularia integra* Macgillivray, 1842	[46,130,140]
*Orthopyxis clytioides* (Lamouroux, 1824)	[40,89]	Santos Bay, Santo Amaro Island and Itanhaém, São Paulo, Brazil	*Orthopyxis sargassicola* (Nutting, 1915)	[1](?), [8](?)
			*Orthopyxis integra* (Macgillivray, 1842)	[13](?)
	[90]	La Coronilla, Rocha, Uruguai	-	-
*Orthopyxis crenata* (Hartlaub, 1901)	[42]	South of Cabo Frio, Brazil	*Orthopyxis crenata* (Hartlaub, 1901)	[97]
			*Orthopyxis sargassicola* (Nutting, 1915)	[1,8,13,31]
*Orthopyxis everta* (Clark, 1976)	[44]	Puerto Madryn, Argentina	*Campanularia* (*Orthopyxis*) *everta* Clark, 1876	[45]
			*Campanularia subantarctica* Millard, 1971	[46,130]
			*Orthopyxis mollis* (Stechow, 1919)	[97]
			*Orthopyxis hartlaubi* El Beshbeeshy, 2011	[138]
			*Campanularia lennoxensis* Jäderholm, 1903	[56]
*Orthopyxis hartlaubi* El Beshbeeshy, 2011	[137,138]	Santa Cruz and Tierra del Fuego, Argentina	*Orthopyxis mollis* (Stechow, 1919)	[97,150]
			*Campanularia hartlaubi* (El Beshbeeshy, 2011)	[56]
*Orthopyxis integra* (Macgillivray, 1842)	[13,53,54,140, 149]	Rio de Janeiro, São Paulo, Paraná and Santa Catarina, Brazil; Beagle Channel	-	-
*Orthopyxis lennoxensis* (Jäderholm, 1903)	[40,89,148]	Santo Amaro and São Sebastião Islands, São Paulo, Brazil	*Orthopyxis crenata* (Hartlaub, 1901)	[42]
			*Orthopyxis sargassicola* (Nutting, 1915)	[1,8,13,31]
*Orthopyxis minuta* Vannucci, 1949	[41]	Brazil, Rio de Janeiro, Francês Island	*Orthopyxis sargassicola* (Nutting, 1915)	[1](?), [8,13]
			*Orthopyxis integra* (Macgillivray, 1842)	[13](?)
*Orthopyxis sargassicola* (Nutting, 1915)	[1,10,13,48,51,54, 55]	Espírito Santo, Rio de Janeiro, São Paulo, Paraná and Santa Catarina, Brazil	-	-

The symbol (?) indicate doubt in the identification, according to the original citations.

Currently, *O. sargassicola* and *O. integra* have been reported to occur in the southwestern Atlantic. In Brazil, *O. sargassicola* was recorded off the coast of Espírito Santo [10,48] and São Paulo states [1,49,50,51], and together with *O. integra*, it has been recorded along the coast of Rio de Janeiro [10,52,53], Paraná [54] and Santa Catarina states [13]. They are usually found in shallow waters, though have also been recorded in deeper areas of 35 and 70 meters [10,53], and frequently occur in epiphytic associations, often on macroalgae of the genus *Sargassum* C.Agardh, 1820 [1,13,50,51,54,55]. The species *O. sargassicola*, for instance, is among the most common and abundant species of hydroids in ephypytic environments in São Paulo and Paraná states [51,54]. In Argentina, *O. caliculata* (accepted as *Campanularia integra*, [46]) was recorded in Puerto Madryn, Chubut [43] and *O. integra* in Punta Peñas, Sán Julian ([46], as *C. integra*); a third species, *O. everta* (Clark, 1876), was recorded by Blanco [44,45] along the coast of Argentina, but it was later re-identified as *C. subantarctica* by Blanco [46] and is now thought to be two different species [47,56] (Table 1). Studies with *Orthopyxis* from Argentina are restricted to their original records, in which species are generally reported in epiphytic or epizoic associations, from shallow waters to depths of 157 meters [43,46]. Species of *Campanularia*, on the other hand, are frequently reported in epizoic associations in Argentina, often occurring on poriferans, bryozoans and abundantly on other hydroids, such as *Amphisbetia operculata* (Linnaeus, 1758) and *Plumularia setacea* (Linnaeus, 1758) [4,57,58, 59]. They are also found on molluscs, gorgonaceans and polychaete tubes, especially in areas where soft bottoms are predominant [6,9]. However, the distribution and substrate associations of *Orthopyxis*, and some species of *Campanularia*, from the southwestern Atlantic are not settled, since there are still many disagreements in the literature regarding the status of species records (Table 1). As well, the taxonomy of *O. integra* and *O. sargassicola*—two species traditionally found in the southwestern Atlantic—remains uncertain, casting doubts on the validity of their records.

Molecular data have been useful for analyzing interspecific boundaries in groups with difficult taxonomies e.g., [60,61,62,63]. For the Hydrozoa, the number of such molecular studies has increased over the last few years, particularly with respect to species delimitation e.g., [64,65,66,67,68,69,70,71,72,73,74] and misidentifications related to incomplete knowledge of morphology and life cycles e.g., [75]. Although there have been relatively few molecular studies involving representatives of the family Campanulariidae e.g., [14,23,24,25,76], these studies have provided important evidence for delimiting species boundaries within this family, suggesting the non-monophyly of Campanulariidae [14,73] and of some species of *Clytia* Lamouroux, 1812 and *Orthopyxis* [14,23,24,25].

The goal of this study was to reassess species boundaries within the genus *Orthopyxis* based on species models from the southwestern Atlantic. Furthermore, morphological characters associated with *Orthopyxis* are re-evaluated, one new species and one new record of *Orthopyxis* are described, and the intergeneric limits of *Orthopyxis* and *Campanularia* are reassessed.

## Materials and Methods

### Study Area and sampled taxa

**Fig 1:**
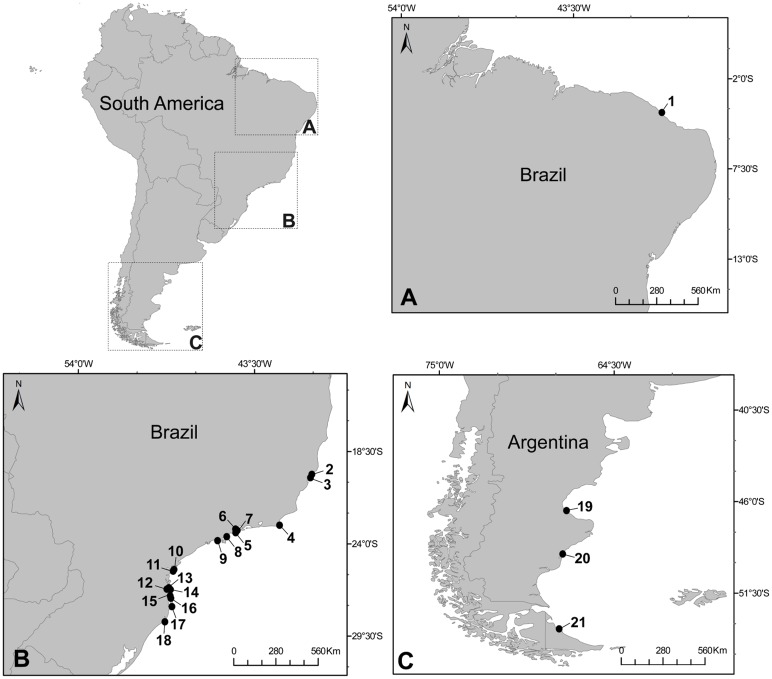
Map of sampling areas in Brazil and Argentina. Circles indicate specific sites were species were sampled. The numbers correspond to the records listed in Table 2.

Specimens of the genus *Orthopyxis* and *Campanularia* were sampled in Brazil and Argentina (Fig. 1, Table 2). Samples were carried out in the northeastern (state of Ceará) and southeastern coast of Brazil (states of Espírito Santo, Rio de Janeiro, São Paulo, Paraná and Santa Catarina), and south of Argentina (provinces of Santa Cruz and Tierra del Fuego). All necessary permits were obtained for the field studies (sampling permits 16802–1 and 16802–2 SISBIO/ICMBio—Instituto Chico Mendes de Conservação da Biodiversidade), and no protected species were sampled. Colonies were collected during low tide on a variety of substrates, including rocks, algae (*Sargassum* sp. and *Macrocystis pyrifera*), mussel shells and other hydroid colonies (mainly species of Sertulariidae), and preserved in 95% ethanol. Species were identified based on taxonomic descriptions [19,31,47,77,78] and, whenever possible, by comparisons with type materials or other reference materials available in museums. Species vouchers were deposited in the Museu de Zoologia da Universidade de São Paulo (MZUSP), Brazil, and in the National Museum of Natural History, Smithsonian Institution (USNM), United States of America (Table 2). One specimen of the Campanulariinae genus *Silicularia* Meyen, 1834 from Argentina was included in several of the analyses because it is thought to be related to *Orthopyxis* cf. [14]. Two species of the genus *Obelia* Péron & Lesueur, 1810 (subfamily Obeliinae, sister group of Campanulariinae according to [14] and [73]) were used as outgroups in the phylogenetic analysis. All sequences were deposited in GenBank (accession numbers in Table 2). Additional data reported in this study (e.g. geographical coordinates, images) were deposited in the National Database Marine Biodiversity (available at https://marinebiodiversity.lncc.br/metacatui/).

**Table 2:** Codes, sampling sites, museum vouchers and GenBank acession numbers for the specimens included in the phylogenetic analyses.

Species	Sampling site and specimen code in tree	Coordinates (number in Fig. 1)	Voucher	GenBank Acession Number		
				16S	COI	ITS
*Obelia dichotoma*	Sandwich Marina, Massachusetts, USA	41°16′15″N 70°15′30″W	MZUSP 1776	KM603472	KM603473	KM603474
*Obelia longissima*	Gloucester State Pier, Massachusetts, USA	42°36′51″N 70°39′06″W	MZUSP 1807	KM603468	KM603470	KM603471
*Orthopyxis crenata*	Caponga (CB), Cascavel, Ceará, Brazil	04°02.348′S 38°11.572′W (1)	MZUSP 2633	KM405590		KM454926
*Orthopyxis sargassicola*	Praia Formosa (FB1), Aracruz, ES, Brazil	Specific coordinate unknown (2)	MZUSP 2629	KM405610	KM405542	KM454946
*Orthopyxis sargassicola*	Praia Formosa (FB2), Aracruz, ES, Brazil	Specific coordinate unknown (2)	MZUSP 2630	KM405611	KM405541	
*Orthopyxis sargassicola*	Praia dos Padres (PB1), Aracruz, Espírito Santo (ES), Brazil	19°55.941′S 40°07.327′W (3)	MZUSP 2617	KM405622	KM405531	KM454957
*Orthopyxis sargassicola*	Praia dos Padres (PB2), Aracruz, ES, Brazil	19°55.941′S 40°07.327′W (3)	MZUSP 2618	KM405623	KM405530	KM454958
*Orthopyxis sargassicola*	Praia dos Padres (PB3), Aracruz, ES, Brazil	19°55.941′S 40°07.327′W (3)	MZUSP 2619	KM405624	KM405529	KM454959
*Orthopyxis sargassicola*	Praia dos Padres (PB4), Aracruz, ES, Brazil	19°55.941′S 40°07.327′W (3)	MZUSP 2620	KM405625	KM405528	KM454960
*Orthopyxis sargassicola*	Praia dos Padres (PB5), Aracruz, ES, Brazil	19°55.941′S 40°07.327′W (3)	MZUSP 2627	KM405626	KM405527	KM454961
*Orthopyxis sargassicola*	Praia dos Padres (PB6), Aracruz, ES, Brazil	19°55.941′S 40°07.327′W (3)	MZUSP 2628	KM405627	KM405526	KM454962
*Orthopyxis sargassicola*	Praia dos Padres (PB7), Aracruz, ES, Brazil	19°55.941′S 40°07.327′W (3)	MZUSP 2632		KM405525	KM454963
*Orthopyxis caliculata*	Praia João Gonçalves (JGB1), Búzios, Rio de Janeiro (RJ), Brazil	Specific coordinate unknown (4)	MZUSP 2612	KM405582		KM454918
*Orthopyxis caliculata*	Praia João Gonçalves (JGB2), Búzios, RJ, Brazil	Specific coordinate unknown (4)	MZUSP 2613	KM405583		KM454919
*Orthopyxis caliculata*	Praia João Gonçalves (JGB3), Búzios, RJ, Brazil	Specific coordinate unknown (4)	MZUSP 2614	KM405584	KM405565	KM454920
*Orthopyxis caliculata*	Praia João Gonçalves (JGB4), Búzios, RJ, Brazil	Specific coordinate unknown (4)	MZUSP 2615	KM405585		KM454921
*Orthopyxis sargassicola*	Paraty (PTY1), RJ, Brazil	Specific coordinate unknown (5)	MZUSP 2605	KM405628	KM405524	KM454964
*Orthopyxis sargassicola*	Paraty (PTY2), RJ, Brazil	Specific coordinate unknown (5)	MZUSP 2606	KM405629	KM405523	KM454965
*Orthopyxis sargassicola*	Paraty (PTY3), RJ, Brazil	Specific coordinate unknown (5)	MZUSP 2607	KM405630	KM405522	KM454966
*Orthopyxis sargassicola*	Paraty (PTY4), RJ, Brazil	Specific coordinate unknown (5)	MZUSP 2608	KM405631	KM405521	KM454967
*Orthopyxis sargassicola*	Paraty (PTY5), RJ, Brazil	Specific coordinate unknown (5)	MZUSP 2609	KM405632	KM405520	KM454968
*Orthopyxis sargassicola*	Ilha dos Ratos (RI), Paraty, RJ, Brazil	23°11.640′S 44°36.408′W (6)	MZUSP 2610	KM405633	KM405519	KM454969
*Orthopyxis sargassicola*	Ilha dos Meros (MI), Paraty, RJ, Brazil	23°11.264′S 44°34.635′W (7)	MZUSP 2611	KM405621	KM405532	KM454956
*Orthopyxis sargassicola*	Praia do Lázaro (LB1), Ubatuba, SP, Brazil	23°30′32.64″S 45°08′18.52″W (8)	MZUSP 2594	KM405612	KM405540	KM454947
*Orthopyxis sargassicola*	Praia do Lázaro (LB2), Ubatuba, SP, Brazil	23°30′32.64″S 45°08′18.52″W (8)	MZUSP 2595	KM405613	KM405539	KM454948
*Orthopyxis sargassicola*	Praia do Lázaro (LB3), Ubatuba, SP, Brazil	23°30′32.64″S 45°08′18.52″W (8)	MZUSP 2596	KM405614	KM405538	KM454949
*Orthopyxis sargassicola*	Praia do Lázaro (LB4), Ubatuba, SP, Brazil	23°30′32.64″S 45°08′18.52″W (8)	MZUSP 2597	KM405615	KM405537	KM454950
*Orthopyxis crenata*	Praia do Lázaro (LB5), Ubatuba, SP, Brazil	23°30′32.64″S 45°08′18.52″W (8)	MZUSP 2598	KM405591		KM454927
*Orthopyxis sargassicola*	Praia do Lázaro (LB6), Ubatuba, SP, Brazil	23°30′32.64″S 45°08′18.52″W (8)	MZUSP 2599	KM405616	KM405536	KM454951
*Orthopyxis sargassicola*	Praia do Lázaro (LB7), Ubatuba, SP, Brazil	23°30′32.64″S 45°08′18.52″W (8)	MZUSP 2600	KM405617	KM405535	KM454952
*Orthopyxis crenata*	Praia do Lázaro (LB8), Ubatuba, SP, Brazil	23°30′32.64″S 45°08′18.52″W (8)	MZUSP 2601	KM405592		KM454928
*Orthopyxis sargassicola*	Praia do Lázaro (LB9), Ubatuba, SP, Brazil	23°30′32.64″S 45°08′18.52″W (8)	MZUSP 2602	KM405618	KM405534	KM454953
*Orthopyxis sargassicola*	Praia do Lázaro (LB10), Ubatuba, SP, Brazil	23°30′32.64″S 45°08′18.52″W (8)	MZUSP 2603	KM405619		KM454954
*Orthopyxis sargassicola*	Praia do Lázaro (LB11), Ubatuba, SP, Brazil	23°30′32.64″S 45°08′18.52″W (8)	MZUSP 2604	KM405620	KM405533	KM454955
*Orthopyxis sargassicola*	Praia Preta, São Sebastião (SS), São Paulo (SP), Brazil	Specific coordinate unknown (9)	MZUSP 2593	KM405634	KM405518	KM454970
*Orthopyxis mianzani*	Praia do Miguel (MB1), Ilha do Mel, Paraná (PR), Brazil	25°33′22.12"S 48°17′55.36"W (10)	MZUSP 2570	KM405602	KM405550	KM454938
*Orthopyxis mianzani*	Praia do Miguel (MB2), Ilha do Mel, PR, Brazil	25°33′22.12"S 48°17′55.36"W (10)	MZUSP 2571	KM405603	KM405549	KM454939
*Orthopyxis mianzani*	Praia do Miguel (MB3), Ilha do Mel, PR, Brazil	25°33′22.12"S 48°17′55.36"W (10)	MZUSP 2572	KM405604	KM405548	KM454940
*Orthopyxis mianzani*	Praia do Miguel (MB4), Ilha do Mel, PR, Brazil	25°33′22.12"S 48°17′55.36"W (10)	MZUSP 2573	KM405605	KM405547	KM454941
*Orthopyxis mianzani*	Praia do Miguel (MB5), Ilha do Mel, PR, Brazil	25°33′22.12"S 48°17′55.36"W (10)	MZUSP 2574	KM405606	KM405546	KM454942
*Orthopyxis mianzani*	Praia de Fora (FOB1), Ilha do Mel, PR, Brazil	25°34′22.58"S 48°18′32.77"W (11)	MZUSP 2575	KM405595	KM405557	KM454932
*Orthopyxis mianzani*	Praia de Fora (FOB2), Ilha do Mel, PR, Brazil	25°34′22.58"S 48°18′32.77"W (11)	MZUSP 2576	KM405596	KM405556	KM454933
*Orthopyxis mianzani*	Praia de Fora (FOB3), Ilha do Mel, PR, Brazil	25°34′22.58"S 48°18′32.77"W (11)	USNM 1259970	KM405597	KM405555	KM454934
*Orthopyxis mianzani*	Praia de Fora (FOB4), Ilha do Mel, PR, Brazil	25°34′22.58"S 48°18′32.77"W (11)	MZUSP 2577	KM405598	KM405554	KM454935
*Orthopyxis mianzani*	Praia de Fora (FOB5), Ilha do Mel, PR, Brazil	25°34′22.58"S 48°18′32.77"W (11)	MZUSP 2578	KM405599	KM405553	KM454936
*Orthopyxis mianzani*	Praia de Fora (FOB6), Ilha do Mel, PR, Brazil	25°34′22.58"S 48°18′32.77"W (11)	MZUSP 2579	KM405600	KM405552	KM454937
*Orthopyxis mianzani*	Praia de Fora (FOB7), Ilha do Mel, PR, Brazil	25°34′22.58"S 48°18′32.77"W (11)	MZUSP 2580	KM405601	KM405551	
*Orthopyxis caliculata*	Praia da Armação (AB), Penha, SC, Brazil	26°47′S 48°37′W (12)	MZUSP 2565	KM405578	KM405567	KM454914
*Orthopyxis caliculata*	Praia da Paciência (PAB1), Penha, Santa Catarina (SC), Brazil	26°46′38″S 48°36′10″W (13)	MZUSP 2550	KM405586	KM405564	KM454922
*Orthopyxis crenata*	Praia da Paciência (PAB2), Penha, SC, Brazil	26°46′38″S 48°36′10″W (13)	MZUSP 2551	KM405593	KM405559	KM454930
*Orthopyxis caliculata*	Praia da Paciência (PAB3), Penha, SC, Brazil	26°46′38″S 48°36′10″W (13)	MZUSP 2552	KM405587	KM405563	KM454923
*Orthopyxis caliculata*	Praia da Paciência (PAB4), Penha, SC, Brazil	26°46′38″S 48°36′10″W (13)	MZUSP 2554	KM405588	KM405562	KM454924
*Orthopyxis caliculata*	Praia da Paciência (PAB5), Penha, SC, Brazil	26°46′38″S 48°36′10″W (13)	MZUSP 2556	KM405589	KM405561	KM454925
*Orthopyxis mianzani*	Praia da Paciência (PAB6), Penha, SC, Brazil	26°46′38″S 48°36′10″W (13)	MZUSP 2559	KM405607	KM405545	KM454943
*Orthopyxis crenata*	Praia da Paciência (PAB7), Penha, SC, Brazil	26°46′38″S 48°36′10″W (13)	MZUSP 2560	KM405594	KM405558	KM454931
*Orthopyxis caliculata*	Praia Grande (GB), Penha, SC, Brazil	26°46′S 48°35′W (14)	MZUSP 2563	KM405581	KM405566	KM454917
*Orthopyxis caliculata*	Praia de Bombas (BB), Bombinhas, SC, Brazil	27^o^07′52.44″S 48°30′49.02″W (15)	MZUSP 4265	KM405579		KM454915
*Orthopyxis caliculata*	Praia da Conceição (COB), Bombinhas, SC, Brazil	27°12′1.26″S 48°29′32.04″W (16)	MZUSP 4177	KM405580		KM454916
*Orthopyxis sargassicola*	Ilha Campeche (CI1), Florianópolis, SC, Brazil	27°41′27″S 48°27′51″W (17)	MZUSP 4597	KM405608	KM405544	KM454944
*Orthopyxis sargassicola*	Ilha Campeche (CI2), Florianópolis, SC, Brazil	27°41′27″S 48°27′51″W (17)	MZUSP 4599	KM405609	KM405543	KM454945
*Orthopyxis crenata*	Prainha, Laguna (LG), SC, Brazil	28°36.097′S 48°48.957′W (18)	MZUSP 5055		KM405560	KM454929
*Orthopyxis* sp. indet.	Caleta Olivia, Argentina	46°25.539′S 67°31.183′W (19)	MZUSP 2644	KM405635		KM454971
Campanulariidae sp. indet.	La Mina, Puerto San Julián (SJ1), Argentina	49°09.413′S 67°37.987′W (20)	MZUSP 2638	KM405576		KM454912
*Campanularia subantarctica*	La Mina, Puerto San Julián (SJ2), Argentina	49°09.413′S 67°37.987′W (20)	MZUSP 2639	KM405574	KM405569	KM454910
Campanulariidae sp. indet.	La Mina, Puerto San Julián (SJ3), Argentina	49°09.413′S 67°37.987′W (20)	MZUSP 2640	KM405577		KM454913
*Campanularia* sp.	La Mina, Puerto San Julián (SJ4), Argentina	49°09.413′S 67°37.987′W (20)	MZUSP 2641	KM405572	KM405571	KM454908
*Campanularia* sp.	La Mina, Puerto San Julián (SJ5), Argentina	49°09.413′S 67°37.987′W (20)	MZUSP 2642	KM405573	KM405570	KM454909
*Campanularia subantarctica*	La Mina, Puerto San Julián (SJ6), Argentina	49°09.413′S 67°37.987′W (20)	MZUSP 2643	KM405575	KM405568	KM454911
*Silicularia rosea*	Río Grande, Cabo Santo Domingo, Argentina	53°41.330′S 67°50.673′W (21)	MZUSP 2645	KM405636		KM454972

### Molecular data

Nuclear DNA and mitochondrial DNA were extracted using Instagene (Bio-Rad Laboratories, Hercules, California, USA), according to the manufacturer’s protocol. Portions of the mitochondrial 16S ribosomal RNA gene and the cytochrome oxidase subunit I (COI) gene as well as the entire nuclear Internal Transcribed Spacer (ITS) region (ITS1, 5.8S ribosomal RNA gene and ITS2) were amplified by PCR and verified on 1.5% agarose gels (PCR conditions and primers are described in Table 3). PCR products were purified using the AMPure purification kit (Agencourt Bioscience Corporation, Beckman Coulter, Beverly, Massachusetts, USA), and purified products were prepared for sequencing using the Big Dye Terminator v3.1 Cycle Sequencing kit (Applied Biosystems, Foster City, California, USA) and the same PCR primers. The sequencing reactions were carried out using an ABI PRISM 3100 Genetic Analyzer (Applied Biosystems, Foster City, California, USA).

**Table 3:** Primers and PCR conditions for DNA amplification.

Genes	Primers	Reference	Primers Sequence (5′-3′)	PCR conditions	Fragment Size (approx.)
16S	C&B1 (F)^1^	[152]	TCGACTGTTTACCAAAAACATAGC	Init. Denat.: 94°C, 3min; 5 cycles: 94°C, 30sec; 45°C, 50sec; 72°C, 1min; 30 cycles: 95°C, 30sec; 50°C, 45sec; 72°C, 1min; Fin. Ext.: 72°C, 5min; 10°C	610 bp
	C&B2 (R)	[152]	ACGGAATGAACTCAAATCATGTAAG		
	2Hydrom (R)	Ale E, LEM^2^	CTGTTATCCCTAAGGTAGC		475 bp
COI	LCO1490 (F)^1^	[153]	-GGTCAACAAATCATAAAGATATTGG-	Init. Denat.: 94°C, 2min; 10 cycles: 94°C, 30sec; 48°C, 1min; 72°C, 1min20sec; 25 cycles: 94°C, 30sec; 50°C, 40sec; 72°C, 1min20sec; Fin. Ext.: 72°C, 7min; 10°C	660 bp
	HCO2198 (R)	[153]	-TAAACTTCAGGGTGACCAAAAAATCA-		
	HCOcato (R)	[117]	-CCTCCAGCAGGATCAAAGAAAG		630 bp
ITS1–5.8S-ITS2	CAS18sF1 (F)	[154]	TACACACCGCCCGTCGCTACTA	Init. Denat.: 94°C, 3min; 35 cycles: 95°C, 30sec; 50°C, 45sec; 72°C, 1min; Fin. Ext.: 72°C, 7min; 4°C	765 bp
	F5′ (F)	[118]	TAACAAGGTTTCCGTAGG		630 bp
	ITS1A (F)	[155]	-GTAACAAGGTTTCCGTAGGTG		630 bp
	CAS28sB1d (R)^1^	[154]	TTCTTTTCCTCCSCTTAYTRATATGCTTAA		
	jfITS1–5F (F)	[116]	-GGTTTCCGTAGGTGAACCTGCGGAAGGATC	Init. Denat.: 94°C, 2min; 35 cycles: 94°C, 30sec; 55°C, 45sec; 72°C, 1min; Fin. Ext.: 72°C, 7min; 4°C	680 bp
	ITS-R-28S-15 (R)	Maronna MM, LEM^2^	ACTCGCCGTTACTAGGGGAATCCTTGTTAG		

(F) Forward (R) Reverse.

^1^Used in conjunction with different forward or reverse primers.

^2^Primers designed by members of the Laboratory of Marine Evolution (LEM), University of Sao Paulo, Brazil.

Sequences were assembled and edited using Geneious (version 7.1 created by Biomatters, Auckland, New Zealand), and aligned using MAFFT [79]. The obtained sequences were compared with those deposited in GenBank using the Basic Local Alignment Search Tool (BLAST, [80]) to confirm genes and species of interest. Additionally, the ITS1 and ITS2 regions were extracted from the complete ITS sequences using the sequence from *Hydra circumcincta* [81] in GenBank (GU722663) as a guide to delimit the ITS1 sequences and the ITS2 Database [82] to delimit the ITS2 sequences. The coding sequences of COI were translated and compared with the complete mitochondrial genome of *Laomedea flexuosa* [83] (GenBank NC_016463) to ensure pseudogenes were not amplified. Since not all sequences of the same marker had the same length (see Table 3), some portions of the longer sequences were excluded from the alignments to adjust all sequences to the same length.

### Phylogenetic analysis

Phylogenetic analyses were performed on (a) individual markers, (b) combined mitochondrial markers (16S+COI), (c) combined nuclear markers (ITS1+ITS2), and (d) the entire combined dataset (16S+COI+ITS1+ITS2), using maximum likelihood (ML) and parsimony (P) criteria. The datasets were built using unique haplotypes, and the combined datasets included only those specimens with sequences available for all markers (details of the analyses in Table 4).

**Table 4:** Details of the datasets used in the phylogenetic analyses.

	Total	16S+COI	ITS1+ITS2	16S	COI	ITS1	ITS2
Number of characters	1553	1046	509	476	575	263	242
Number of informative sites (P)	665	261	390	113	153	214	163
Number of most parsimonious trees (P)	74	116	4115	3	11	5	2130
Minimum length (P)	1276	511	1056	284	304	623	365
Model of nucleotide evolution (ML)	GTR+G	GTR+I+G	GTR+G	GTR+I	GTR+G	GTR+G	SYM+G

(P) Parsimony, (ML) Maximum Likelihood.

Sequences of nuclear DNA with ambiguous sites (17 ITS1 and 22 ITS2 sequences) were treated using IUPAC ambiguity codes. The maximum number of ambiguous sites recorded for one sequence was five (the ITS2 sequence of a specimen from Penha, Santa Catarina), and 46% of the sequences had only one ambiguous site. Sequences with identical IUPAC codes at identical positions were considered as the same haplotype in the analyses.

Phylogenetic analyses using parsimony (P) criteria were performed using the PAUP 4.0b10 [84] and TNT [85] programs. Analyses consisted of 1000 unweighted heuristic searches using a random algorithm and branch-swapping using the TBR (tree bisection-reconnection) algorithm. Gaps were considered as a fifth state. Branch support was estimated in TNT with bootstrapping on 1000 replicates. Phylogenetic analyses using Maximum Likelihood (ML) criteria were performed using PALM (Phylogenetic Reconstruction by Automatic Likelihood Model Selector, [86]) with the most appropriate model of nucleotide evolution for each dataset based on *Akaike Information Criterion* (AIC, Table 4). Branch support was estimated with bootstrapping on 1000 replicates. Phylogenetic *p*-distances (uncorrected) were calculated using the PAUP 4.0b10 program.

### Morphological analysis

We performed Principal Component Analysis (PCA, [87]) on a correlation matrix based on 37 different measures of the trophosome (S1 Table) of the voucher specimens of *O. caliculata* and *O. mianzani* sp. nov. (the same specimens used in the phylogenetic analyses). For both species, we did not include any characters from the gonothecae in the PCA, as not all colonies presented this reproductive structure. This analysis was performed to better delimitate the species by assessing the degree of variation for their morphological characters and by identifying their most relevant diagnostic characters.

### Nomenclatural acts

The electronic edition of this article conforms to the requirements of the amended International Code of Zoological Nomenclature, and hence the new names contained herein are available under that Code from the electronic edition of this article. This published work and the nomenclatural acts it contains have been registered in ZooBank, the online registration system for the ICZN. The ZooBank LSIDs (Life Science Identifiers) can be resolved and the associated information viewed through any standard web browser by appending the LSID to the prefix “http://zoobank.org/”. The LSID for this publication is: urn:lsid:zoobank.org:pub:280AC2D0–9DCE-4BCE-AF85–2586B3951522. The electronic edition of this work was published in a journal with an ISSN, and has been archived and is available from the following digital repositories: PubMed Central and LOCKSS.

## Results

**Fig 2:**
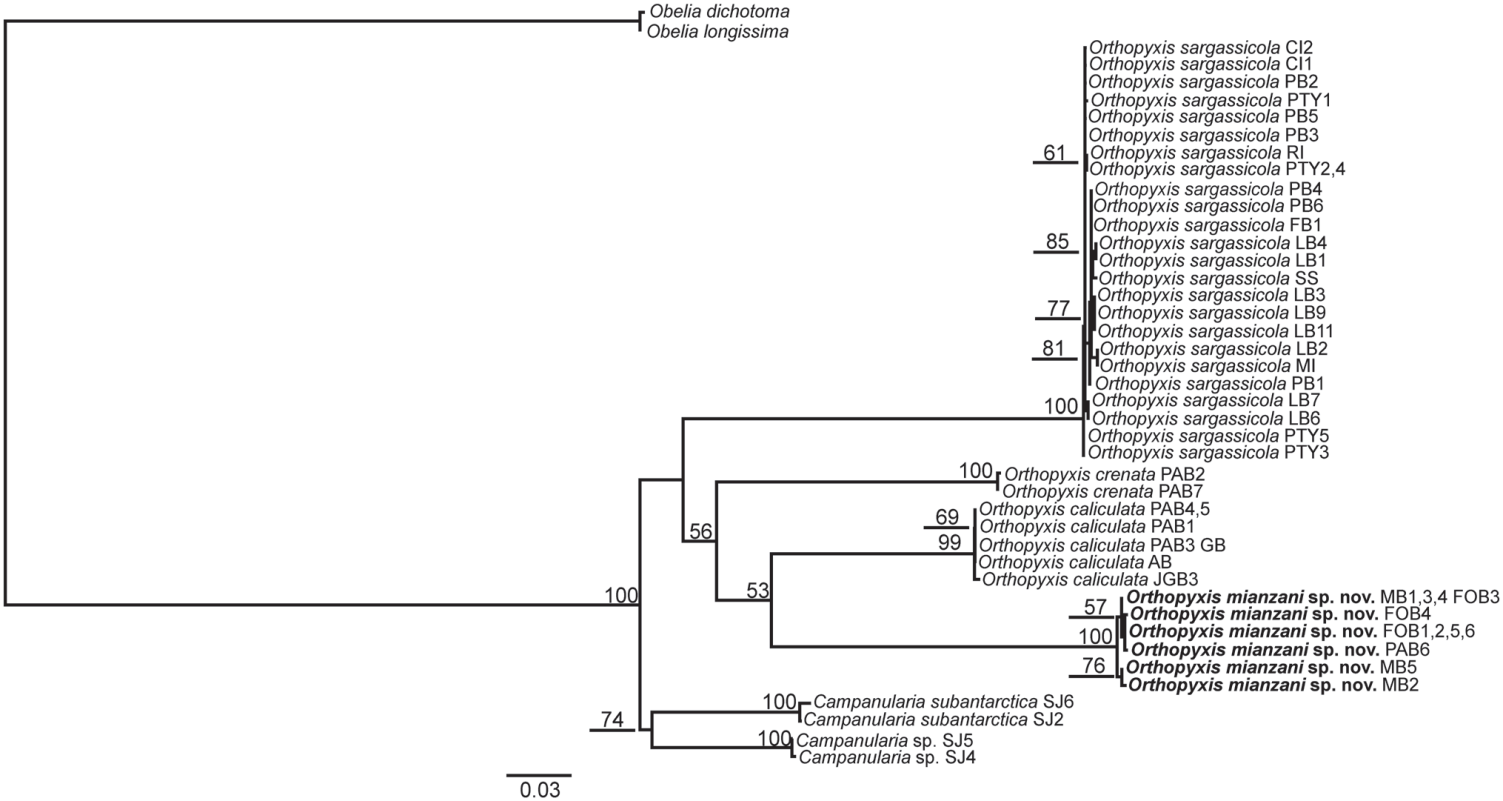
Maximum Likelihood tree based on 16S, COI, ITS1 and ITS2 data. Bootstrap values are shown for each node. Nodes without numbers indicate support below 50.

**Fig 3:**
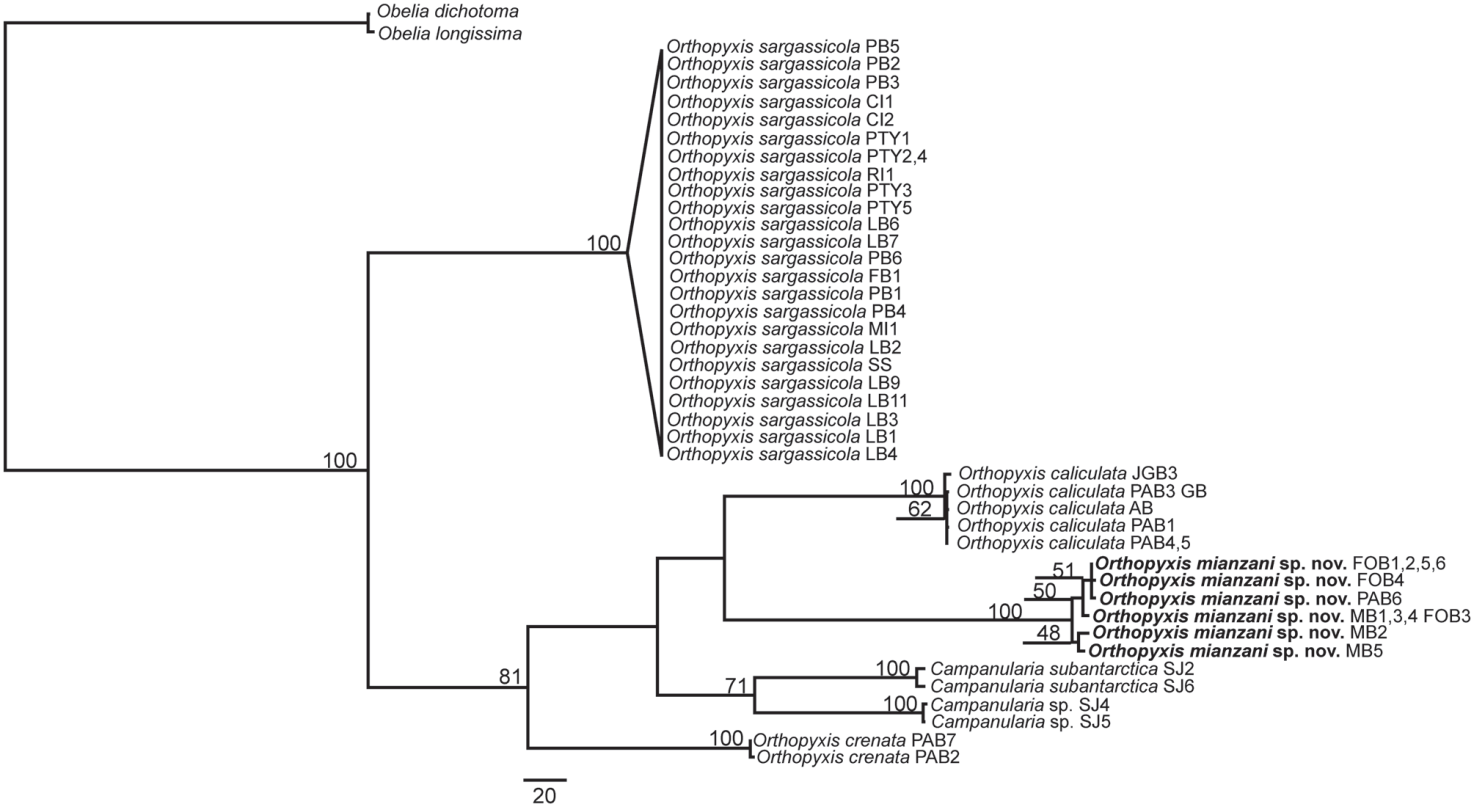
One of the 74 most parsimonious trees based on 16S, COI, ITS1 and ITS2 data. These trees are only different in the position of the haplotypes within *O. sargassicola* clade, which is collapsed. Bootstrap values are shown for each node. Nodes without numbers indicate support below 50.

**Fig 4:**
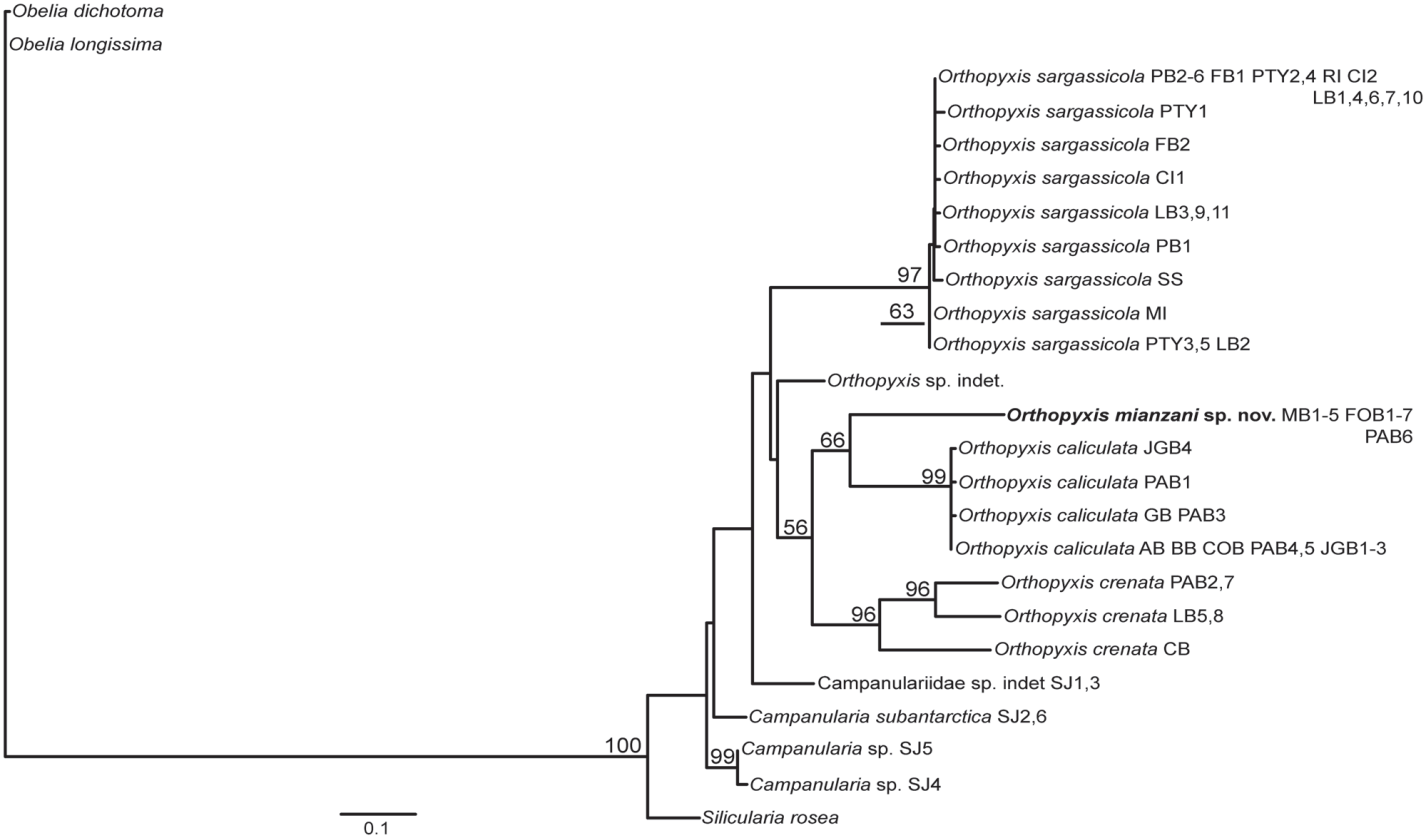
Maximum Likelihood tree based on 16S data. Bootstrap values are shown for each node. Nodes without numbers indicate support below 50.

**Fig 5:**
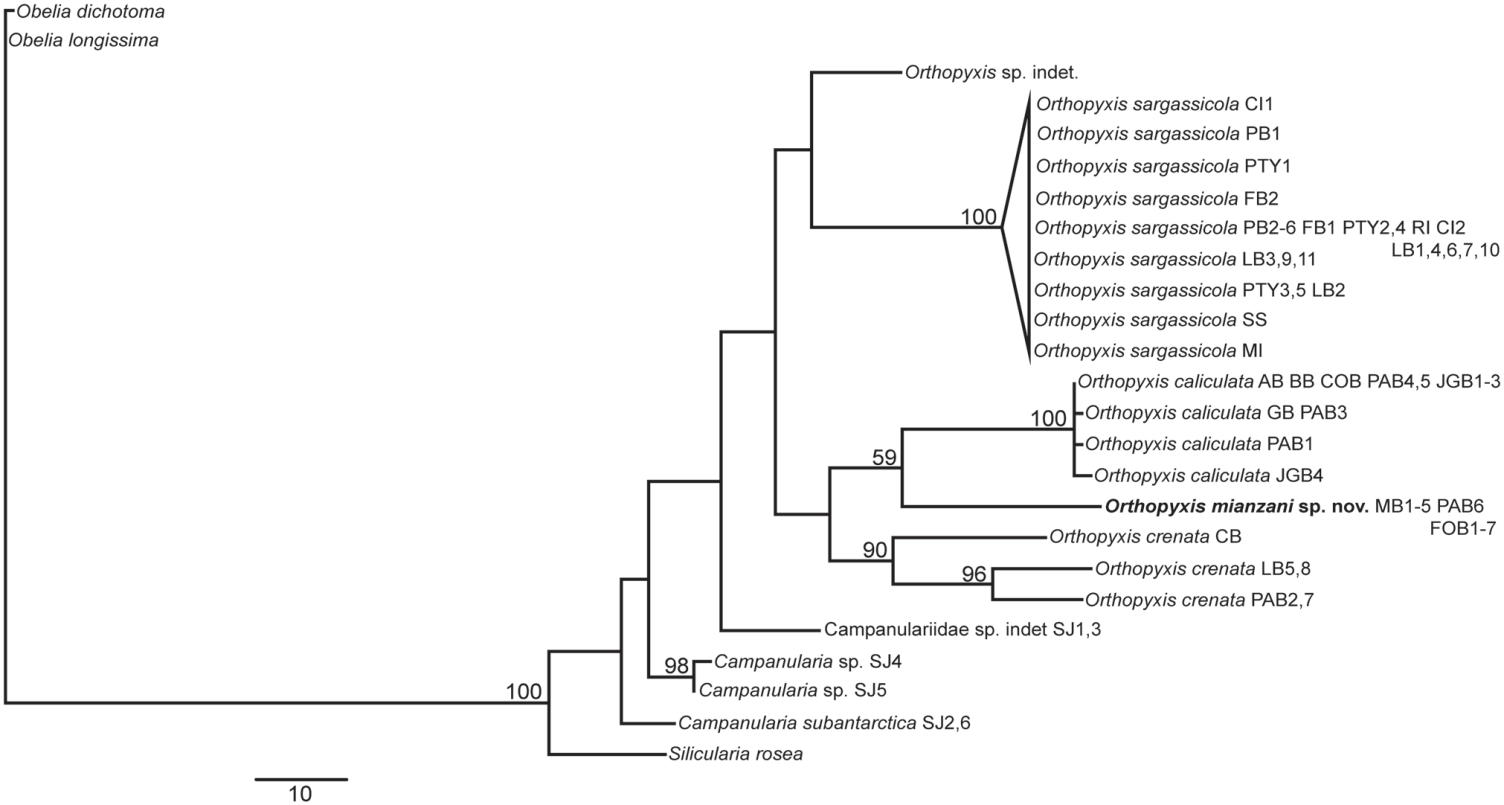
One of the three most parsimonious trees based on 16S data. These trees are only different in the position of the haplotypes within *O. sargassicola* clade, which is collapsed. Bootstrap values are shown for each node. Nodes without numbers indicate support below 50.

Nearly all the topologies obtained using the different datasets identified six well-defined clades with high branch support values. However, these topologies did present some incongruencies with respect to the phylogenetic relationships among these clades. The individual and combined nuclear datasets showed low resolution and low values for branch support, whereas the combined mitochondrial datasets showed higher resolution but also had low branch support (S1–S10 Figs.). The combined dataset involving all four markers revealed the best definition of the relationships among the lineages, with a higher frequency of well supported nodes (all six less inclusive clades with bootstrap = 99–100, Figs. 2–3). In addition, the 16S topologies showed the most congruent results (Figs. 4–5). Therefore the topologies involving the combined and the 16S datasets represented the most robust hypothesis for our data and are used as our working hypothesis for discussions.

### The genera *Orthopyxis* and *Campanularia*

The genus *Orthopyxis* was monophyletic according to the 16S topologies and the ML topology with the combined dataset, although with low support value (bootstrap<50, Figs. 2, 4–5). *Orthopyxis* was not monophyletic in the P topology with the combined dataset, in which species assigned to *Campanularia* fell within *Orthopyxis* as a sister group to *Orthopyxis caliculata* (Hincks, 1853)+*Orthopyxis mianzani* sp. nov. (Fig. 3). Although not conclusive, *Orthopyxis* was monophyletic in the majority of our topologies, a hypothesis we follow in this study. However, this hypothesis requires further testing with the addition of more representatives from the genus *Campanularia*.

*Campanularia* was monophyletic only in topologies derived from the combined dataset. One of the lineages of *Campanularia* corresponds morphologically to *Campanularia subantarctica* Millard, 1971, and it is characterized by the deep hydrothecae with bluntly rounded marginal teeth, subhydrothecal spherule present; gonothecae oval-elongated arising from hydrorhiza, with distal aperture on top of a low collar [77,88], ([47], as *C. lennoxensis*). The second lineage of *Campanularia* is also morphologically similar to *C. subantarctica*, but we were unable to identify this lineage to the species level due to the lack of gonothecae. Additionally, these two lineages showed genetic distances of up to 7.83% for mitochondrial markers and 26.38% for nuclear markers (Table 5), indicating that they likely represent two distinct species.

**Table 5:** Minimum and maximum *p*-distances (uncorrected) (%) from the mithocondrial dataset (low left corner) and nuclear dataset (up right corner).

Species	*Orthopyxis sargassicola*	*Orthopyxis caliculata*	*Orthopyxis mianzani*	*Orthopyxis crenata*	*Campanularia subantarctica*	*Campanularia* sp.	Campanulariidae sp. indet.	*Orthopyxis* sp. indet.
*Orthopyxis sargassicola*	**0.35/0.51**	17.51–44.05	19.05–43.19	9.66–41.07	13.69–45.42	18.39–44.10	22.02–40.72	12.30–36.72
*Orthopyxis caliculata*	8.68–12.87	**0.30/0.00**	18.62–27.86	13.22–31.76	9.84–29.57	14.74–25.30	15.81–28.67	15.38–23.61
*Orthopyxis mianzani*	9.33–16.17	7.81–15.65	**0.17/1.28**	14.90–35.54	17.87–28.60	24.10–24.73	19.98–33.19	17.70–29.36
*Orthopyxis crenata*	9.33–13.39	8.68–13.74	9.33–15.48	**4.43/3.31**	7.81–10.96	7.81–10.61	17.13–34.58	2.52–32.52
*Campanularia subantarctica*	7.38–9.74	6.72–11.65	8.24–14.78	11.19–38.43	**0.69/0.51**	12.80–26.38	16.26–38.79	13.13–33.48
*Campanularia* sp.	7.38–9.74	6.72–10.09	7.81–14.78	13.06–40.55	2.60–7.83	**0.10/0.00**	19.25–33.31	14.66–27.52
Campanulariidae sp. indet.	7.38–8.03	7.16–7.38	8.24	7.38–8.03	4.77	4.77	**0.00**	17.57–30.13
*Orthopyxis* sp. indet.	5.86–6.51	6.07–6.29	8.24	7.38–8.46	4.56	5.21	3.69	**0.00**

Values in the diagonal indicate mean intraspecific distances (mithocondrial/nuclear markers).

### Species of the genus *Orthopyxis*

We delimited four lineages of the genus *Orthopyxis* in the southwestern Atlantic, three of which correspond morphologically to *Orthopyxis sargassicola* (Nutting, 1915), *Orthopyxis crenata* (Hartlaub, 1901), and *Orthopyxis caliculata* (Hincks, 1853) (considered a synonym of *Orthopyxis integra* (Macgillivray, 1842) by some authors; see discussion below), and one of which is new to science (Figs. 2–5). These species showed genetic distances ranging from 7.81–16.17% and 9.66–44.05% for mitochondrial and nuclear makers, respectively (Table 5).

**Fig 6:**
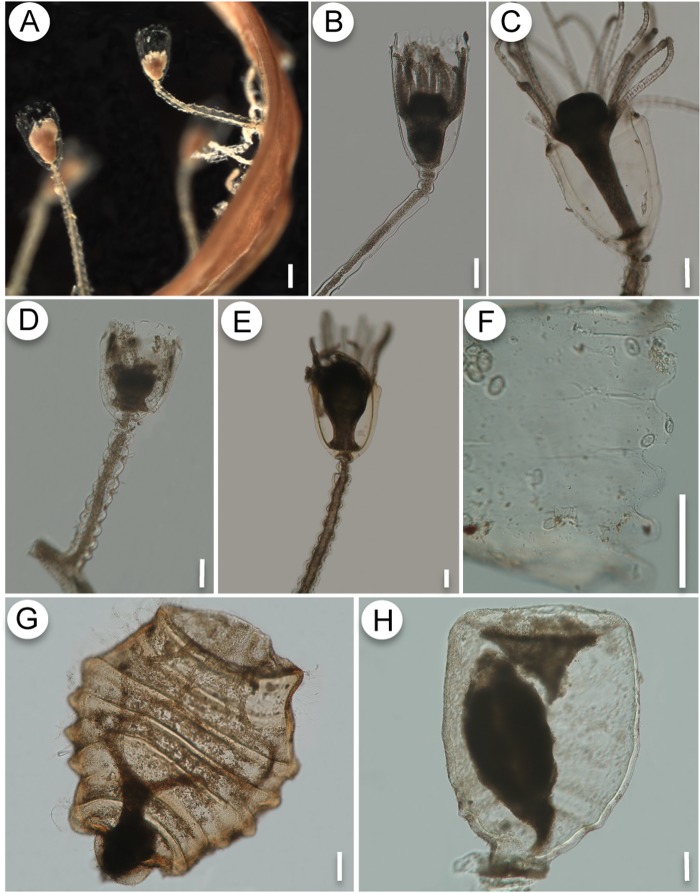
A-C, G: *Orthopyxis sargassicola*. A: general view of the colony on *Sargassum* sp.; B-C: detail of the trophosome, showing variation in perisarc thickness of hydrotheca; G: gonotheca. D-F, H: *Orthopyxis crenata*. D-E: detail of the trophosome; F- detail of the hydrothecal cusps; H- gonotheca. Scales: A—200 μm; B-H—100 μm.

The specimens of *O. sargassicola* and *O. crenata* recorded here have the general features of *Orthopyxis*, such as a thick perisarc (variable to some extent), campanulate hydrothecae, sinuous pedicels, and subhydrothecal spherule (Fig. 6). The presence of rounded hydrothecal cusps and a laterally compressed, completely ribbed gonotheca, are distinctive characters of *O. sargassicola* [1,13,31] (Fig. 6A-C, G), whereas *O. crenata* is characterized by low, rounded hydrothecal cusps and laterally compressed, smooth gonothecae [29,47] (Fig. 6D-F, H). Although these species can be readily distinguished by comparing their gonothecae, morphological variation in the size and shape of the hydrothecal cusps may cause these diagnostic characters to overlap when the gonotheca is absent, hampering identification. This is the first record of *Orthopyxis crenata* in the southwestern Atlantic, although previous authors may have overlooked this species due to its morphological similarity with *O. sargassicola*.

The species *O. caliculata* and *O. mianzani* sp. nov., although highly genetically divergent (Table 5), have similar morphologies that could be traditionally associated with *Orthopyxis integra* (Macgillivray, 1842). Both species have stolonal colonies, sinuous pedicels, subhydrothecal spherule, campanulate hydrotheca with rim even, and gonotheca roughly cylindrical, with wide aperture, truncated on top [19,78]. However, they are morphologically distinct with respect to characters usually assumed to show wide intraspecific variation, such as perisarc thickness and length of the hydrothecae and pedicels.

**Fig 7:**
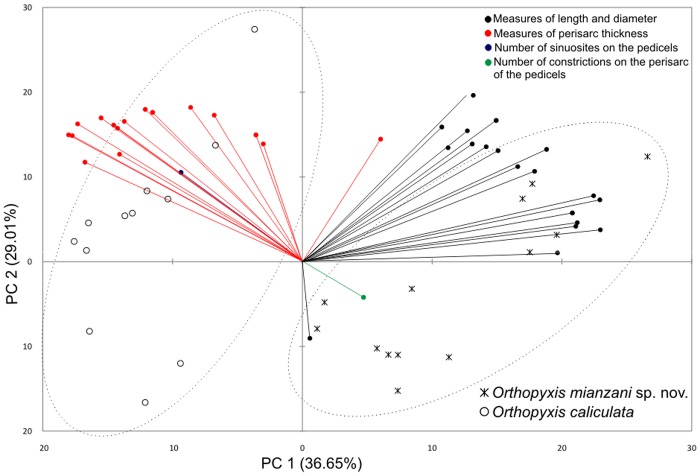
Correlation biplot of the first and second principal components of the PCA based on morphometric variables of *Orthopyxis caliculata* and *Orthopyxis mianzani* sp. nov. from the southwestern Atlantic. The percentage of variation explained by each principal component is shown in parentheses.

PCA performed using the morphometric data for *O. caliculata* and *O. mianzani* sp. nov. (Fig. 7) showed that the two lineages are clearly separated by perisarc thickness and polyp general dimensions. Specimens of *O. caliculata* have a thicker perisarc and smaller general dimensions (length and diameter of the hydrothecae, pedicels, and subhydrothecal spherule) of the polyp (Fig. 7). These results show that, although variable to some extent, perisarc thickness and polyp dimensions can be used to delimitate these species. Therefore, we believe the name *Orthopyxis caliculata* (Hincks, 1853) is the correct identification of one of these lineages, and we corroborate the validity of that species.

### Systematic Account

***Orthopyxis caliculata* (Hincks, 1853)**

(Fig. 8)

**Fig 8:**
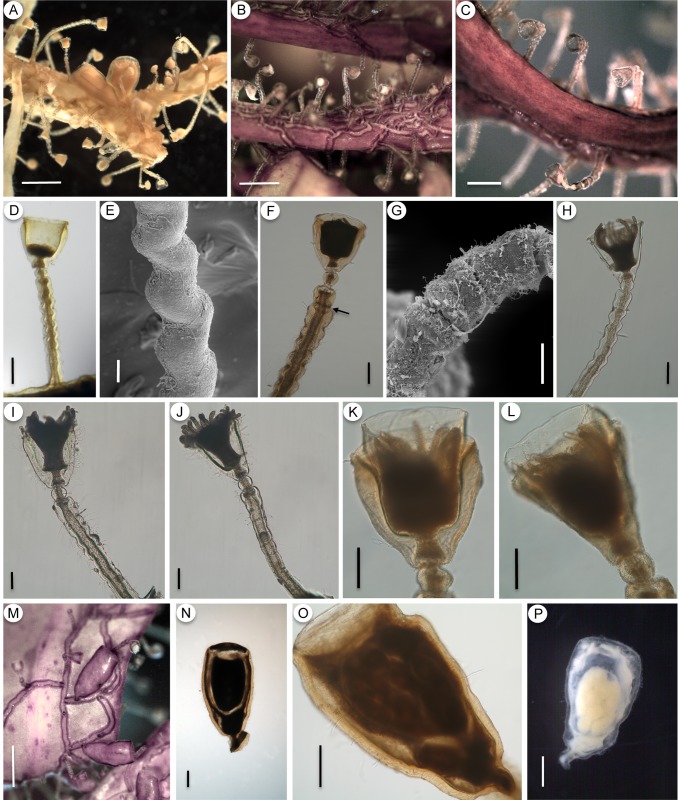
*Orthopyxis caliculata*. A-C: general view of the colony (A-MZUSP 4177; B,C- MZUSP 1563); D-H: detail of the trophosome with the sinuosities of the pedicel (E) and constrictions in the perisarc (arrow in F, G) (D-MZUSP 2550; E-MZUSP 2565; F-MZUSP 2554; G-MZUSP 4177; H-MZUSP 2552); I-J: positions of maximum (I) and minimum (J) perisarc thickness of the trophosome (MZUSP 2615); K-L: detail of the hydrotheca, showing two different forms due to compression (MZUSP 2554); M: general view of gonothecae on algae (MZUSP 2563); N: detail of male gonotheca (MZUSP 2554); O-P: detail of female gonothecae (O-MZUSP 2563; P-MZUSP 2613). Scales: A,B,M—1 mm; C—500 μm; D,F,H,O—200 μm; E—20 μm; G—50 μm; I,J,K,L—100 μm; N,P—300 μm.

*Orthopyxis clytioides*—Vannucci-Mendes, 1946 [40]: 546, Est.1, Figs. 6,7.—Vannucci, 1951 [89]: 111 [not *Orthopyxis clytioides* (Lamouroux, 1824)].

*Orthopyxis minuta* Vannucci, 1949 [41]: 234, t.1, Figs.15–17, t.2, Fig.18.—Vannucci, 1951 [89]: 108. (syn. nov.)

**Fig 9:**
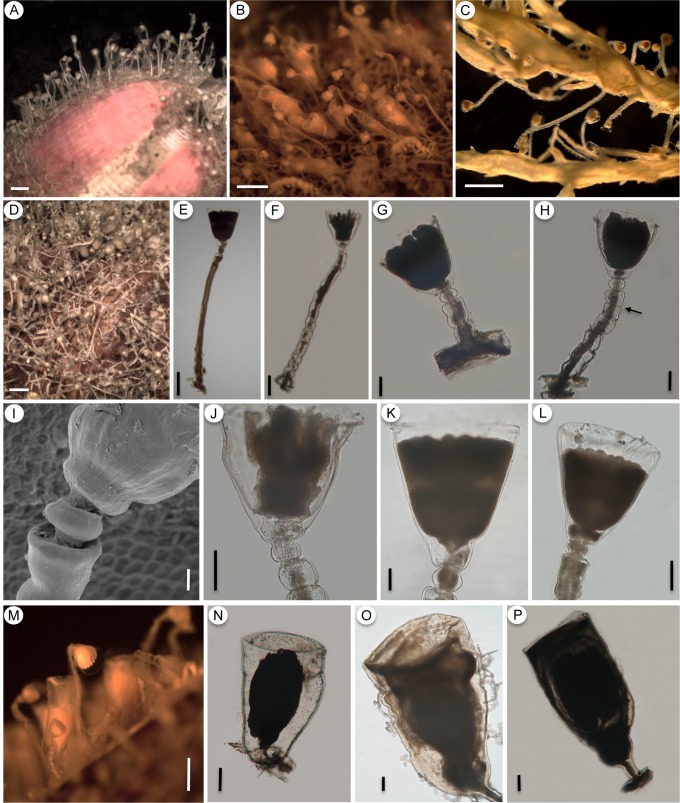
*Orthopyxis mianzani* sp. nov. A-C: general view of the colony (A-MZUSP 2575; B-MZUSP 2580; C-MZUSP 2559); D: detail of the hydrorhiza (USNM 1259970); E-H: details of the trophosome, showing variation in pedicels from smooth (E) to sinuous (F), variation in the length of the pedicels (compare E, F and G) and constrictions of the perisarc (H, arrow) (E-MZUSP 2576; F-MZUSP 2572; G-MZUSP 2570; H-MZUSP 2574); I: detail of subhydrothecal spherule (USNM 1259970); J-L: detail of hydrothecae (J-MZUSP 2572; K-MZUSP 2576; L-MZUSP 2579); M: general view of the gonotheca on natural substrate (MZUSP 2580); N-P: detail of female gonothecae (N-MZUSP 2572; O-USNM 1259970; P-MZUSP 2580). Scales: A-D—1 mm; E—300 μm; F, N, P—200 μm; G, H, J-L, O—100 μm; I—20 μm; M—500 μm.

*Orthopyxis caliculata*—Blanco, 1964 [43]: 157, L.1, Figs. 4,9.

*Orthopixis clytioides*—Milstein, 1976 [90]: 77, Figs. 8,9,11 [not *Orthopyxis clytioides* (Lamouroux, 1824)].

*Campanularia integra*—Blanco, 1994 [46]: 192 [not *Campanularia integra* Macgillivray, 1842].

*Orthopyxis integra*—Miranda et al., 2011 [13]: 347, Fig. 25a-d [not *Orthopyxis integra* (Macgillivray, 1842)].

**Material examined. Brazil**, Santa Catarina (SC), Penha, Praia Grande, 26°46’S 48°35’W, 0–1 m, 08.vii.2009, with female gonothecae, on algae, coll. E.C. Bornancin, **MZUSP 2563**; SC, Penha, Praia da Paciência, 26°46’38”S 48°36’10”W, 3 m, 02.vii.2009, without gonothecae, on algae, coll. A.F. Cunha, **MZUSP 2550**; SC, Penha, Praia da Paciência, 26°46’38”S 48°36’10”W, 0–1 m, 02.vii.2009, without gonothecae, on algae, with some colonies of *Obelia* sp., coll. A.F. Cunha, **MZUSP 2552**; SC, Penha, Praia da Paciência, 26°46’38”S 48°36’10”W, 3 m, 01.vii.2009, with male gonothecae, on algae, coll. A.F. Cunha, **MZUSP 2554**; SC, Penha, Praia da Paciência, 26°46’38”S 48°36’10”W, 3 m, 01.vii.2009, without gonothecae, on algae, coll. A.F. Cunha, **MZUSP 2556**; SC, Penha, Praia da Armação, 0–1 m, 07.vii.2009, without gonothecae, on algae, coll. E.C. Bornancin, **MZUSP 2565**; SC, Bombinhas, Praia de Bombas, 27°07’52.44”S 48°30’49.02”W, 0–2 m, 03.xii.2006, with female gonothecae, on algae, coll. A.C. Marques & T.P. Miranda, **MZUSP 4265**; SC, Bombinhas, Praia da Conceição, 27°12’1.26”S 48°29’32.04”W, 0–2 m, 02.xii.2006, with male and female gonothecae (two colonies), on algae, coll. A.C. Marques, E. Ale, M.A. Imazu & T.P. Miranda, **MZUSP 4177**; Rio de Janeiro, Búzios, Praia de João Gonçalves, coordinate unknown, 20.viii.2009, with few female gonothecae, on algae, coll. L.S. Miranda, A.C. Morandini & S.N. Stampar, **MZUSP 2612**, **MZUSP 2613**, **MZUSP 2614** and **MZUSP 2615**.

**Additional material examined. Argentina**, Chubut, Puerto Madryn, *Orthopyxis caliculata* (Hincks, 1853), O.M. Blanco det., Museo de La Plata, **MLP 47** to **MLP 54**; Santa Cruz, San Julián, Punta Peñas, *Orthopyxis caliculata* (Hincks, 1853), O.M. Blanco det., **MLP 55**; Santa Cruz, Punta Peñas, *Campanularia integra* Macgillivray, 1842, O.M. Blanco, det., **MLP 8536**. **Uruguay**, Rocha, La Coronilla, *Orthopixis clytioides* (Lamouroux, 1824) [incorrect subsequent spelling], det. A. Milstein. **United States**, Alaska, Aleutian Islands, *Orthopyxis integra* (Macgillivray, 1842), A. Govindarajan det., National Museum of Natural History, **USNM 1106184**. **Kara Sea**, *Campanularia integra* Macgillivray, 1842, **USNM 17834**.

**Description**. Colonies stolonal, up to 1.6 mm high. Hydrothecae and pedicels laterally compressed, amount of compression varying according to perisarc thickness. Pedicels arise from creeping, flattened hydrorhiza at irregular intervals. Hydrorhiza with very thick perisarc (31–47.5 μm). Pedicels sinuous, with 5–13 sinuosities (crenations) throughout their length, forming a “zig-zag” on pedicels, not spiral, as commonly assumed (Fig. 8E). Occasionally 1–4 constrictions, usually on upper portion of pedicels (most likely regions of growth) (Fig. 8F-G). Pedicels 588–1260 μm in length, usually with thick perisarc (23.54 μm on average) but also colonies with thinner perisarc occur (11.5–30 μm, Fig. 8H). Subhydrothecal spherule present immediately below hydrotheca, slightly smaller than pedicel in diameter, with thick perisarc (14–32.5 μm). Hydrotheca campanulate, 230–374 μm in length, rim smooth, sometimes slightly everted (Fig. 8H) and occasionally growing beyond the thick hydrothecal walls (Fig. 8K-L). Hydrotheca laterally compressed, more conspicuous when perisarc is very thick. Hydrotheca may show two different forms in relation to the compression: (1) when viewed from its broader aspect (i.e., position of maximum perisarc thickness), hydrotheca with thick, straight and parallel walls, gradually projecting inwards towards base, where the perisarc reaches maximum thickness and forms an interior chamber, in which the hydranth rests (Fig. 8K); (2) when viewed from its narrower aspect (i.e., position of minimum perisarc thickness), the much thinner walls are oblique, tapering towards the base (Fig. 8L). Hydranth with 22–26 tentacles. Male and female gonothecae with similar morphology, up to 1.2 mm high, arising from hydrorhiza on short, smooth pedicels, usually growing parallel to substrate. Young gonothecae short and conical, truncated on top, with wide aperture; mature gonothecae with walls oblique at base but gradually elongating and straightening to become parallel, upper portion also truncated, with wide aperture. Gonothecae laterally compressed, perisarc thick (25–46 μm), with somewhat wavy outline, sometimes more pronounced (Fig. 8M-P). Gonophore with two medusa buds, inferior one smaller, superior one larger, and developing gonads in longitudinal rows.

**Remarks**. *Orthopyxis caliculata* (Hincks, 1853) has been considered a synonym of *O. integra* (Macgillivray, 1842) by many authors. Levinsen [91] was likely the first to assign Hincks’ species to *O. integra* (as *Campanularia integra*), arguing that he possessed colonies of *O. integra* that presented intermediate characters from both species, referring in particular to the thickness of the perisarc of the hydrothecae and the presence of annulations on the gonothecae. Many subsequent authors followed this proposal [19,29,78,92,93,94,95,96,97], also arguing that the characters used to distinguish these species are actually intraspecific variations of the same character.

Hincks [98] noted the shape of the hydrothecae and the presence of a “double cup” and “double” pedicel as the main characters that distinguish *O. integra* and *O. caliculata*. He subsequently amended his description by arguing that the appearance of a “double” hydrotheca and pedicel is a result of the considerable perisarc thickening in this species [26]. The widely accepted notion that these characters represent variations within the same species has prevented many authors from accepting them as informative (as stated above), although some authors who agree with Hincks [26,98] in regarding *O. integra* and *O. caliculata* as separate species point out characters such as the size and shape of the hydrothecae and gonothecae, as important differences between these species e.g., [27,28,99,100]. Indeed, the name *O. caliculata* is currently used as a valid name in some studies [101, 102], based on similar opinions.

Neither species was originally described with gonothecae [98,103], although subsequent descriptions of these species represented the gonothecae of *O. integra* as clearly different from those of *O. caliculata*. The gonothecae of *O. integra* is described as cylindrical, completely spirally grooved throughout, and truncated on top, whereas the gonothecae of *O. caliculata* is described as smooth, oval-elongated, laterally compressed, also truncated on top, and with a wide aperture [26,28,99]. Authors who advocate the synonymy of *O. integra* and *O. caliculata* consider both types of gonothecae as variations within *O. integra* (see [78]). Despite this, Millard [29] notes that she never recorded polyps of *O. integra* in South Africa with spirally grooved gonothecae, and many other records of *O. integra* include only specimens with oval-elongated, smooth gonothecae e.g., [13,97,104,105,106]. Indeed, cylindrical, spirally grooved gonothecae appear to be restricted to northern records of *O. integra* e.g., [38,107,108,109,110], as noted by Bale [99].

We studied non-type material of *O. integra* that presented spirally grooved gonotheca (USNM 17834 from Kara Sea, and 1106184 from Alaska, Aleutian Islands)—in contrast with the oval-elongated, smooth gonotheca of our material—and we have concluded that these two types of gonotheca indicate two different species. These non-type materials of *O. integra* also differ from our specimens of *O. caliculata* in the thickness of the perisarc of the hydrothecae and pedicels, as well as in the length of the hydrothecae, which is larger in *O. integra* (see comparisons on Table 6). Many of these differences have already been noted and discussed by Bale [99], and more recently by Calder et al. [102]. Our molecular analysis revealed two different lineages presenting the traditional morphological characters associated to *O. integra*. A re-evaluation of the morphological characters of these two lineages demonstrates that their most consistent differences rely on characters previously considered to be intraspecific variations by many authors. Therefore, we conclude that the two completely different gonothecae morphologies should not be considered as variations within *O. integra*.

**Table 6:** Comparative measurements of *Orthopyxis caliculata*, *Orthopyxis mianzani* (mean±standard error [range]) and specimens of *Orthopyxis integra* from the National Museum of Natural History, Smithsonian Institution.

Measurements (μm)	*Orthopyxis caliculata* (Np = 12; Ng = 5)	*Orthopyxis mianzani* (Np = 13; Ng = 4)	O. integra** (Np = 3; Ng = 4)	O. integra*** (Np = 4; Ng = 4)
Total length of the trophosome	1213.83±81.58 [840–1658]	1566.77±156.01 [600–2380]	2082.98±197.57 [1695.38–2343.28]	3949.79±718.48 [2437.92–5605.39]
**Hydrorhiza**				
Diameter	84.17±3.37 [65–100]	88.77±3.22 [75–114]	135.29±5.77 [126.08–145.92]	139.80±8.66 [116.24–157.08]
Perisarc thickness	39.92±2.24 [31–47.5]	24.46±1.17 [12.5–30.5]	*	*
**Pedicel**				
Length	825.08±70.62 [588–1260]	943.15±127.76 [190–1870]	1405.38±223.40 [959.41–1652.07]	3337.79±677.82 [1938.89–4958.59]
Diameter	95.71±4.53 [68.5–118]	108±4.15 [89–145]	99.14±7.32 [85.40–110.39]	90.80±5.36 [82.96–106.55]
Perisarc thickness	23.54±1.75 [11.5–30]	11±0.60 [7.5–12.5]	10.32±0.61 [9.01–10.86]	8.31±0.64 [6.97–9.65]
Maximum number of sinuosities	7.97±0.80 [5–13]	4.29±0.76 [0–12]	*	0 (all pedicels smooth troughout)
**Subhydrothecal spherule**				
Length	63.30±3.43 [48–78]	70.69±5.41 [50–120]	74.13±4.94 [65.64–82.75]	52.09±7.81 [33.74–68.08]
Diameter	84.55±2.45 [72–93]	101±3.74 [85–130]	100.80±5.15 [91.28–108.96]	78.63±8.40 [55.14–93.26]
Perisarc thickness	22.35±1.53 [14–32.5]	14.69±1.09 [7.5–22.5]	12.49±2.29 [7.95–15.32]	6.17±0.68 [5.03–8.13]
**Hydrotheca**				
Length	318.33±11.85 [230–374]	418.69±17.74 [328–520]	667.51±22.46 [622.58–690.37]	604.02±56.53 [448.80–717.15]
Diameter at rim	283.17±5.63 [263–312]	369.54±14.97 [304–490]	420.87±4.93 [414.50–430.58]	500.03±25.76 [452.88–569.57]
Diameter at base	157.83±5.90 [120–175]	173.19±2.85 [160–200]	180.33±12.37 [168.41–205.06]	237.48±31.33 [174.94–322.64]
Length:Diameter ratio	1.26±0.04 [0.96–1.60]	1.44±0.04 [1.22–1.71]	1.78±0.05 [1.72–1.88]	1.44±0.16 [0.98–1.65]
Perisarc thickness	29.46±2.22 [15.25–36.5]	7.75±0.80 [2.5–12.5]	9.27±1.32 [7.12–11.66]	4.87±0.79 [2.95–6.23]
**Hydranth**				
Number of tentacles	24±0.58 [22–26] (N = 10)	32.46±5.31 [23–43]	*	*
**Gonotheca**				
Length	1166.42±30.75 [1096–1262.5]	1210±64.16 [1090–1390]	1422.12±96.79 [1202.46–1651.57]	2086.71±87.53 [1933.13–2278.53]
Maximum Diameter	650.33±31.48 [552–772]	722.50±16.52 [690–760]	522.97±19.92 [474.07–571.46]	620.34±15.58 [590.20–659.85]
Length:Diameter ratio	1.82±0.07 [1.57–2.09]	1.79±0.07 [1.66–1.99]	2.74±0.26 [2.10–3.17]	3.37±0.20 [2.95–3.86]
Perisarc thickness	39.21±3.24 [25–46]	21.25±1.25 [20–25]	*	*
**Nematocysts**				
Microbasic mastigophores A type (LenghtxDiam.)	5.14±0.06 [4–6] x 1.80±0.04 [1.5–3] (N = 60)	5.13±0.06 [5–6] x 1.79±0.05 [1.5–2] (N = 40)	*	*
Microbasic mastigophores B type (LengthxDiam.)	10.21±0.11 [9–12] x 2.89±0.05 [2–4] (N = 60)	10.24±0.08 [9–11] x 2.81±0.04 [2.5–3] (N = 40)	*	*

Np = number of polyps measured; Ng = number of gonothecae measured (N = when different number). The measures of diameter and perisarc thickness were obtained from the position of maximum perisarc thickness (broad view).

*Information not obtained.

**USNM17834.

***USNM1106184.

Considering this, we believe that none of the species described in this study, nor the records included in our synonym, should be assigned to *O. integra*; instead, they should be assigned to *O. caliculata*. We understand that it is not simple to delimit these two species morphologically; therefore, we did not include in the synonymy materials we could not access. The one exception is Vannucci’s material [40,41], which is most likely lost (see [1]), for which we tentatively attribute the specimens she described with gonothecae to *O. caliculata*. The specimen *Campanularia integra* recorded by Blanco [43] consists of only one microslide with one polyp without gonothecae, and the hydrotheca of this specimen differs from the typical hydrotheca of *O. caliculata*, being more elongated and cylindrical, similar to the hydrothecae of many species of the genus *Campanularia*. It is unclear whether this morphology is a preparation artifact or an actual morphological difference, so we therefore decided not to include this record in the synonym of *O. caliculata*, pending more detailed study. However, the specimens of *C. integra* recorded by Blanco [46] correspond to the description of *O. caliculata*. Milstein [90] described specimens with gonothecae that also correspond to *O. caliculata*. The records of *O. integra* by Miranda et al. [13] came from localities very close to our records of *O. caliculata*, and examination of their material leaves no doubt that it should be assigned to *O. caliculata*.

**Type locality**. Pegwell Bay, England [98].

**Records from the southwestern Atlantic. Brazil**, São Paulo, Santos Bay, Santo Amaro Island, Itanhaém [40,89]; Rio de Janeiro, Francês Island [41,89], and Búzios (this study); Santa Catarina, Penha (this study) and Bombinhas [13] (and this study). **Uruguay**, Rocha, La Coronilla [90]. **Argentina**, Chubut, Puerto Madryn [43], Santa Cruz, San Julián and Punta Peñas [46].

***Orthopyxis mianzani*** Cunha, Genzano & Marques **sp. nov**. urn:lsid:zoobank.org:act:A6F4A8FB-FDCC-4BE9–8368–6BFE29CAECC4

(Fig. 9)

?*Orthopyxis integra*—Grohmann et al., 2011 [53]: 195, Fig. 3F, 1–4 [not *Orthopyxis integra* (Macgillivray, 1842)].

**Material examined. Holotype: Brazil**, Paraná (PR), Ilha do Mel, Praia de Fora, 25°34’22.58”S 48°18’32.77”W, 0–1 m, 27.vii.2010, with female gonothecae, on mussel shell and cirriped, coll. E.C. Bornancin & A.F. Cunha, **MZUSP 2580**; **Paratypes**: PR, Ilha do Mel, Praia do Miguel, 25°33’22.12”S 48°17’55.36”W, 0–1 m, 26.vii.2010, without gonothecae, on mussel shell, coll. E.C. Bornancin & A.F. Cunha, **MZUSP 2571**, **MZUSP 2573**; with female gonothecae, **MZUSP 2572**, **MZUSP 2574**; without gonothecae, on mussel shell and cirriped, **MZUSP 2570**; PR, Ilha do Mel, Praia de Fora, 25°34’22.58”S 48°18’32.77”W, 0–1 m, 27.vii.2010, without gonothecae, on mussel shell and cirriped, coll. E.C. Bornancin & A.F. Cunha, **MZUSP 2575**, **MZUSP 2579**; with female gonothecae, **USNM 1259970**; without gonothecae, on mussel shell, **MZUSP 2576**; without gonothecae, on *Phragmatopoma* sp., **MZUSP 2577**; without gonothecae, on cirriped, **MZUSP 2578**; Santa Catarina, Penha, Praia da Paciência, 26°46’38”S 48°36’10”W, 0–1 m, 05.vii.2009, without gonothecae, on algae, coll. A.F. Cunha, **MZUSP 2559**.

**Etymology**. This species is named after Dr. Hermes W. Mianzan (CONICET and Instituto Nacional de Investigación y Desarollo Pesquero—INIDEP, Mar del Plata, Argentina) for his dedication and commitment to the study of South American cnidarians, and his leadership towards the integration of Latin American marine scientists. Unfortunately, our great “amigo” Hermes passed away during the writing of this manuscript.

**Diagnosis**. Hydrothecae, pedicels and gonothecae with thin perisarc. Lateral compression only on gonothecae, nearly no compression detectable on hydrothecae or pedicels, both usually longer when compared with other species of *Orthopyxis*. Reduced amount of sinuosities on pedicels, sometimes almost completely smooth. Gonothecae smooth and different from other *Orthopyxis* species with ribbed gonothecae.

**Description**. Colonies stolonal, up to 2.3 mm high. Gonothecae laterally compressed but rarely hydrothecae (compression better observed in hydrothecae with thicker perisarc). Pedicels arise from creeping, flattened hydrorhiza at irregular intervals. Hydrorhiza with moderately thick perisarc (12.5–30.5 μm) and large (diameter 75–114 μm, Fig. 9D). Pedicels usually with slight sinuosities at base and smooth throughout their length, sometimes either sinuous throughout (up to 12 tenuous sinuosities) (Fig. 9F) or with 1–4 marked perisarc constrictions at upper portion (Fig. 9H). Pedicels usually long, rarely small, 190–1870 μm in length, with moderately thick perisarc (7.5–12.5 μm). Subhydrothecal spherule present right below hydrotheca, slightly smaller than pedicel in diameter, with moderately thick perisarc (7.5–22.5 μm). Hydrotheca campanulate, 328–520 μm in length, rim smooth. Perisarc thickness is poorly correlated with hydrothecal form, although hydrotheca may be slightly compressed when perisarc is thicker. Hydrothecal walls slightly oblique with moderately thick perisarc, tapering towards base where perisarc reaches its maximum thickness, forming an interior chamber in which the hydranth rests (Fig. 9J-L). Hydranth with 23–43 tentacles. Female gonothecae up to 1.39 mm high, arising from hydrorhiza on short, smooth pedicels. Young gonotheca short, conical, truncated on top, with wide aperture; mature gonotheca with rounded walls at base, gradually elongating and straightening until parallel, truncated on top, with a wide aperture (Fig. 9N-P). Gonothecae laterally compressed, with moderately thick perisarc (20–25 μm) and a somewhat wavy outline. Gonophore with two medusa buds, inferior one smaller, superior one larger and developing gonads in longitudinal rows.

**Remarks**. Although this species resembles several nominal species of *Orthopyxis*, it presents important morphological differences. With respect to the trophosome, it resembles that of the widely known *Orthopyxis integra* (Macgillivray, 1842), but they differ significantly in gonothecae shape (see remarks of *O. caliculata*; also see [26,28,111]). The gonothecae of *O. mianzani* sp. nov. is also very similar to that of *O. caliculata* (Hincks, 1853), but the length of the pedicels and hydrothecae in *O. mianzani* sp. nov. is 100 μm greater (on average) compared with *O. caliculata* (Table 6; Fig. 7), and its perisarc is, on average, two to three times thinner than that of *O. caliculata* (Table 6; Figs. 8 and 9).

Indeed, a thin perisarc is a good diagnostic character for this species, as it does not appear to be as variable as in other species of *Orthopyxis*. Although there is some variation in perisarc thickness (2.5–12 μm on hydrothecae, 7.5–22.5 μm on subhydrothecal spherule and 7.5–12.5 μm on pedicels), it is never as thick as in *O. caliculata* or as described and illustrated for many other species of *Orthopyxis*, such as *Orthopyxis pacifica* Stechow, 1919, *Orthopyxis angulata* Bale, 1914 (see also [101]) and *Orthopyxis compressima* (Kubota & Yamada, 1992). Even among species currently considered to be synonyms of *O. integra* [19], the perisarc is frequently described as very thick or variable in thickness (e.g., *Orthopyxis compressa* Clark, 1877; *Orthopyxis asymmetrica* Stechow, 1919); in cases where the species is represented with a thin perisarc, other characters appear to differ from those of *O. mianzani* sp. nov., such as the gonothecae (e.g., *Campanularia integriformis* Marktanner-Turneretscher, 1890, *Orthopyxis wilsoni* Bale, 1914).

The slightly sinuous pedicels of *O. mianzani* sp. nov. may also prove to be a good diagnostic character, particularly for distinguishing this species from *O. caliculata*, as these sinuosities are never so marked as in the latter species. This character also differentiates *O. mianzani* sp. nov. from *Orthopyxis clytioides* (Lamouroux, 1824). The pedicels of *O. clytioides*, represented by Lamouroux [112] as real annulations, are quite different from the sinuosities found in *O. mianzani* sp. nov. and other species of *Orthopyxis*, such as *O. integra* and *O. caliculata* [26,28,78]. *Orthopyxis clytioides*, however, still has a doubtful taxonomic status and some authors suggest it may be related to the genus *Obelia* [19,99].

The specimens belonging to *O. integra* recorded by Grohmann et al. [53] in Rio de Janeiro, Brazil, closely resemble this new species, particularly with respect to the thin perisarc and shape of hydrothecae and gonothecae. They are tentatively assigned here to *O. mianzani* sp. nov., pending future study of the material of Grohmann et al. [53].

**Type locality**. Ilha do Mel, Paraná, Brazil.

**Other records from the southwestern Atlantic. Brazil**, Santa Catarina, Penha (this study), Rio de Janeiro [53].

*Silicularia*, *Orthopyxis* sp. indet., and Campanulariidae sp. indet.

*Silicularia rosea* Meyen, 1834 and unidentified specimens were only included in the 16S phylogenies, as we were unable to amplify COI fragments from these specimens. In the 16S phylogenies, *Silicularia rosea* has a basal position relative to the other genera. The highly supported clade *Silicularia*+*Campanularia*+*Orthopyxis* corroborates the close relationships between these genera, although this may have been affected by using a relatively distant root species (*Obelia dichotoma*, *O. longissima*).

The specimens from San Julián, Argentina (Campanulariidae sp. indet.) are morphologically similar to *Orthopyxis mianzani* sp. nov., but their ambiguous position among the different phylogenies (Figs. 4–5; S3–S4, S7–S10 Figs.) makes it difficult to determine their true identity. Considering only the 16S phylogenies, they occupied a basal position among *Orthopyxis*. The specimen from Caleta Olivia, Argentina (*Orthopyxis* sp. indet.) is morphologically similar to *Orthopyxis crenata*, but it lacks gonothecae, which would have allowed for better comparisons, and it also had an ambiguous position in the phylogenies, hampering its identification. This specimen, however, was consistently positioned among the species of the genus *Orthopyxis*. As reliable information for the identification of these specimens was lacking, they were left unidentified until more information is available to determine their taxonomic status.

## Discussion

Our results reinforce the importance of using mitochondrial markers, particularly the 16S rRNA gene, for phylogenetic inferences at many taxonomic levels. The use of 16S to define genera and species is common in studies with the Hydrozoa [24,25,64,66,67,69,76,113], and its potential for barcoding has been demonstrated [71,114]. The resolution levels provided by this gene are also adequate for phylogenetic inferences among putative superfamilies, orders and even subclasses e.g., [65], including the Hydroidolina [16]. In this study, the phylogenetic signal from 16S proved crucial for defining the relationships among the species and genera in these analyses, corroborating the monophyly of the genus *Orthopyxis* and delimiting the four species that occur in the southwestern Atlantic.

By contrast, the nuclear ITS markers are not often used for phylogenetic inferences in studies of the Hydrozoa e.g., [81,115], being more common in studies of the Scyphozoa [116,117,118,119]. Species of the genus *Aurelia* [116,119] and many other invertebrates [120] (Insecta), [121] (Decapoda), [122] (Anthozoa) show great variability in the ITS region, and as a consequence, the ITS markers are generally considered inadequate for supraspecific phylogenetic inferences e.g., [121]. Our ITS analyses corroborate the results obtained with the mitochondrial markers by identifying the same six clades in nearly all analyses. However, the high genetic distance values of the ITS region (Table 5) provide important evidence that phylogenetic information based on ITS on more inclusive levels of the trees is inadequate.

Many molecular studies have characterized cryptic lineages, such as in the genera *Aurelia* (7–9 lineages with genetic distances of 13–24% for COI and 7.8–14.5% for 16S [116,118]) and *Tamoya* (2 lineages with genetic distances of 4.4–4.5% for COI and 2.1–2.5% for 16S [123]). Similar results were obtained for species of the genera *Coryne*, *Turritopsis* and *Cordylophora*, in which interspecific distances ranged from 12.35–15.3% for COI and 3.7–9.2% for 16S [67,69,72]. The genetic distances among the species *O. sargassicola*, *O. crenata*, *O. caliculata* and *O. mianzani* sp. nov. agree with those studies, ranging from 12.35–16% for COI and 7.81–10.2% for 16S. It is important to note, however, that specimens with the diagnostic features of the species *O. integra*, which are commonly reported in the study region, represented two different lineages, neither of which was diagnosed as *O. integra* after a reexamination of their morphological characters. Additionally, the commonly recorded species *C. subantarctica* appears to include two different lineages, although we could not assess the taxonomic status of these lineages due to the low number of specimens. The discovery of different lineages, sometimes in presumably cosmopolitan species, has been recurrent in the family Campanulariidae [14,23,76] and even in genera with extensive revisions aiming to establish interspecific limits (e.g., *Obelia* [18,20]).

Although it is possible to assess species boundaries in the genus *Orthopyxis* using molecular methods, this task is not straightforward using morphological characters, primarily due to wide intraspecific variation. Molecular studies involving morphologically variable groups reveal that morphological characters used to delimit species are frequently misinterpreted, and some traditional diagnostic characters are proving to be inadequate e.g., [124,125]. Despite this, many misleading assumptions regarding the variability of morphological characters in the genus *Orthopyxis* still remain, and conclusions are frequently based on partial or non-formal analyses, derived either from the study of relatively few specimens or from repetition of the opinions of different authors, which are sometimes not based on actual voucher specimens. Indeed, this appears to be the case for the species *O. integra* in the southwestern Atlantic. The intraspecific variation of *O. integra* has been widely documented [19,29,78,91,92,109], and this species is traditionally assumed to be cosmopolitan [19,28,97], but it is clear that the amplitude of intraspecific variation of certain *O. integra* morphological characters has been overestimated. Perisarc thickness, for instance, is an important diagnostic character for the species of *O. caliculata* and *O. mianzani* sp. nov. delimited in this study, although this character is frequently considered too variable to be relevant for diagnostic purposes [19,29,39,47]. Furthermore, we believe that other characters, such as the presence of annulations on the gonothecae, may also be useful diagnostic characters for different lineages within *O. integra* and that they should be investigated more closely. A worldwide revision of *O. integra* is particularly timely, as it appears many of its synonyms may in fact represent true species.

*Orthopyxis sargassicola*, a species widely known in the western Atlantic [1,13,28,31], also appeared as one of the lineages of *Orthopyxis* delimited here. We recorded this species along the southeastern coast of Brazil, and it is known to occur in different regions along the Brazilian coast e.g.,[1,8,13]. There are no records of *O. sargassicola* in Argentina. Other records are from the Gulf Stream (type locality, [28]), east of cape Hatteras [108], and in Aruba, Bonaire and Curaçao [126]. *Orthopyxis crenata*, another lineage delimited in this study, is first recorded for the southwestern Atlantic. Previous records attributed to this species (Table 1) are misidentifications or still have a doubtful taxonomic status. Specimens of *O. crenata* were recorded for Brazil in the states of Ceará (Fortaleza), São Paulo (Ubatuba) and Santa Catarina (Penha and Laguna); other global records include Chile [47,127], New Zealand [39,97,128], South Africa [29] (as *Campanularia crenata*) and Japan [100] (as *C. crenata*). There have been many discussions of the variability of the hydrothecal cusps of *O. crenata*, which vary from slight crenations on the margin of the hydrotheca to well-developed cusps [19,29,39,47,97,100], commonly overlapping with the morphology of the cusps of the species *O. sargassicola*. Calder [31] highlighted the morphological similarities between these two species, which are distinguished by the presence of annulations on the gonothecae of *O. sargassicola*, and by their absence in *O. crenata*. Migotto [1] also noted that some of the specimens he identified as *O. sargassicola* from São Sebastião (SP), Brazil, had morphological similarities to *O. crenata*, particularly with respect to the hydrothecal cusps and medusoids. Neither species can be identified with any certainty in the absence of gonothecae, and therefore, the records of *O. sargassicola* without gonothecae in the southwestern Atlantic should be considered with caution.

Specimens assigned to the genus *Campanularia* here are morphologically similar to the species of *Orthopyxis*, from which they can be distinguished by gonothecae morphology. With respect to the trophosome, the specimens of *Campanularia* do not possess a thickened perisarc on the hydrotheca and pedicels, as is observed in many species of *Orthopyxis*. Galea et al. [47] considered *Campanularia subantarctica* Millard, 1971 to be a synonym of the species *Campanularia lennoxensis* Jäderholm, 1903 based on the argument that their specimens presented gonothecae features found in both species and that perisarc thickness is a variable feature in the Campanulariidae. As already discussed, Campanulariidae is well known for its morphological variability e.g., [19], but we show that perisarc thickness may be a relevant character for delimiting certain species, at least when included in a detailed analysis with a wide range of specimens. Additionally, descriptions of *C. subantarctica* for the study area resemble the specimens described by Millard [77] (e.g., with a thinner perisarc [88,129]). Considering this, we believe the proposed synonymy is premature without more complete evidence, and we regard *C. subantarctica* Millard, 1971 as a valid species, pending more detailed study.

The difficulties in identifying species of *Orthopyxis* and *Campanularia* in the study area are noteworthy, particularly considering the high number of nominal species described and the uncertain synonymies e.g., [1,6,13,31,40,41,42,43,44,45,46,130]. Our analysis corroborates the monophyly of *Orthopyxis* and delimits four species in the southwestern Atlantic, consistent with an assessment of their morphological characters. These findings are crucial to our understanding of the intergeneric limits and species boundaries in the family Campanulariidae. We believe that this integrative approach clarifies many taxonomic difficulties associated with the species of *Orthopyxis*, and we hope that it may serve as a model for the delimitation of other species within the Campanulariidae.

## Supporting Information

S1 Fig A strict consensus of the 116 most parsimonious trees based on 16S and COI data. Bootstrap values are shown for each node. Nodes without numbers indicate support below 50.
(TIF)

S2 Fig Maximum Likelihood tree based on 16S and COI data. Bootstrap values are shown for each node. Nodes without numbers indicate support below 50.
(TIF)

S3 Fig A strict consensus of the 4115 most parsimonious trees based on ITS1 and ITS2 data. Bootstrap values are shown for each node. Nodes without numbers indicate support below 50.
(TIF)

S4 Fig Maximum Likelihood tree based on ITS1 and ITS2 data. Bootstrap values are shown for each node. Nodes without numbers indicate support below 50.
(TIF)

S5 Fig A strict consensus of the 11 most parsimonious trees based on COI data. Bootstrap values are shown for each node. Nodes without numbers indicate support below 50.
(TIF)

S6 Fig Maximum Likelihood tree based on COI data. Bootstrap values are shown for each node. Nodes without numbers indicate support below 50.
(TIF)

S7 Fig A strict consensus of the 5 most parsimonious trees based on ITS1 data. Bootstrap values are shown for each node. Nodes without numbers indicate support below 50.
(TIF)

S8 Fig Maximum Likelihood tree based on ITS1 data. Bootstrap values are shown for each node. Nodes without numbers indicate support below 50.
(TIF)

S9 Fig A strict consensus of the 2130 most parsimonious trees based on ITS2 data. Bootstrap values are shown for each node. Nodes without numbers indicate support below 50.
(TIF)

S10 Fig Maximum Likelihood tree based on ITS2 data. Bootstrap values are shown for each node. Nodes without numbers indicate support below 50.
(TIF)

S1 Table Morphological measures included in the Principal Component Analysis. (DOCX)
