# Supplementary figures and images for: Reassessment of Morphological Diagnostic Characters and Species Boundaries Requires Taxonomical Changes for the Genus Orthopyxis L. Agassiz, 1862 (Campanulariidae, Hydrozoa) and Some Related Campanulariids
Source: PLoS One. 2015 Feb 27;10(2):e0117553. doi: 10.1371/journal.pone.0117553 (PMC4344204; doi:10.1371/journal.pone.0117553)

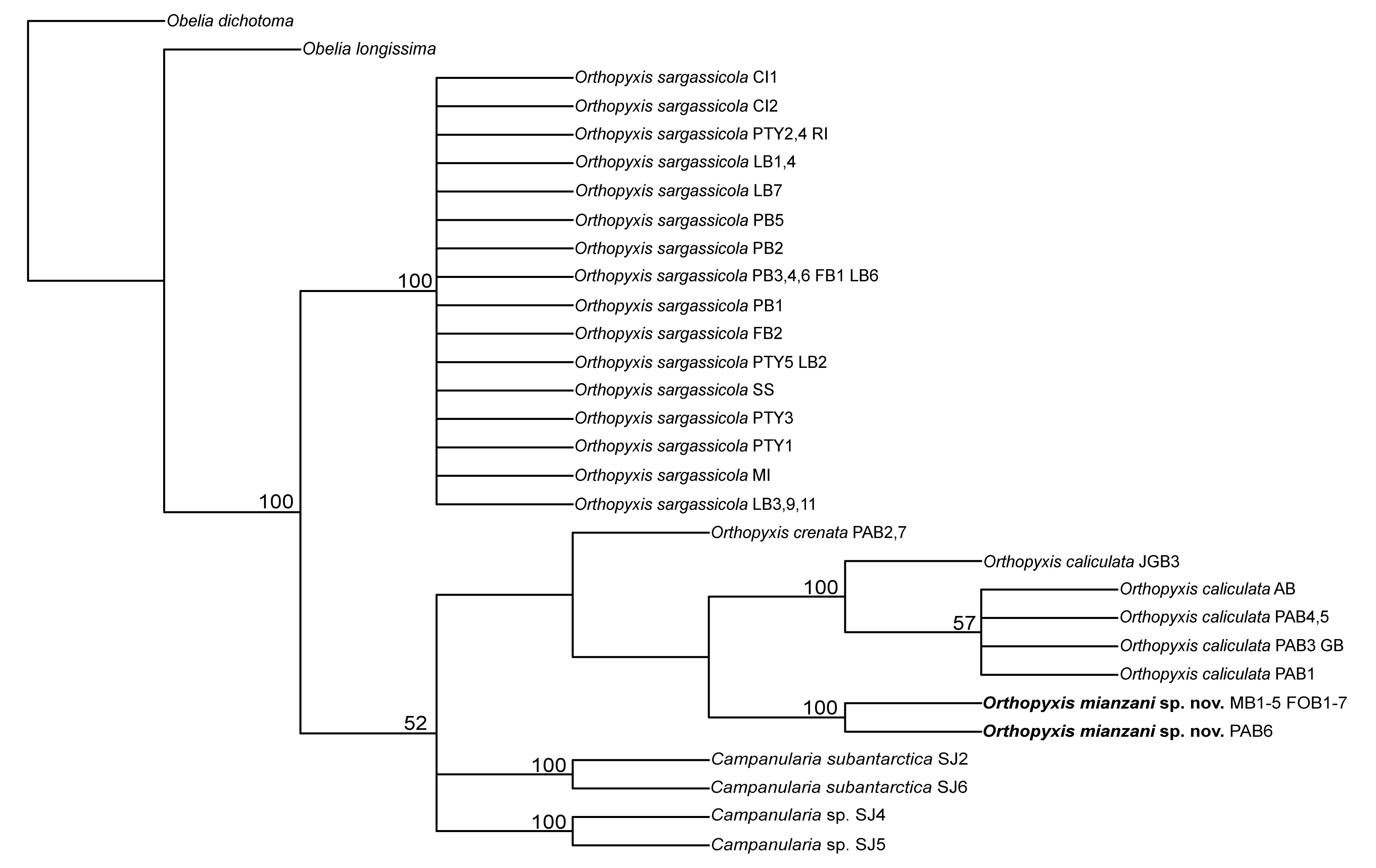

Supplement: S1 Fig — Bootstrap values are shown for each node. Nodes without numbers indicate support below 50. (TIF) [file pone.0117553.s001.tif]

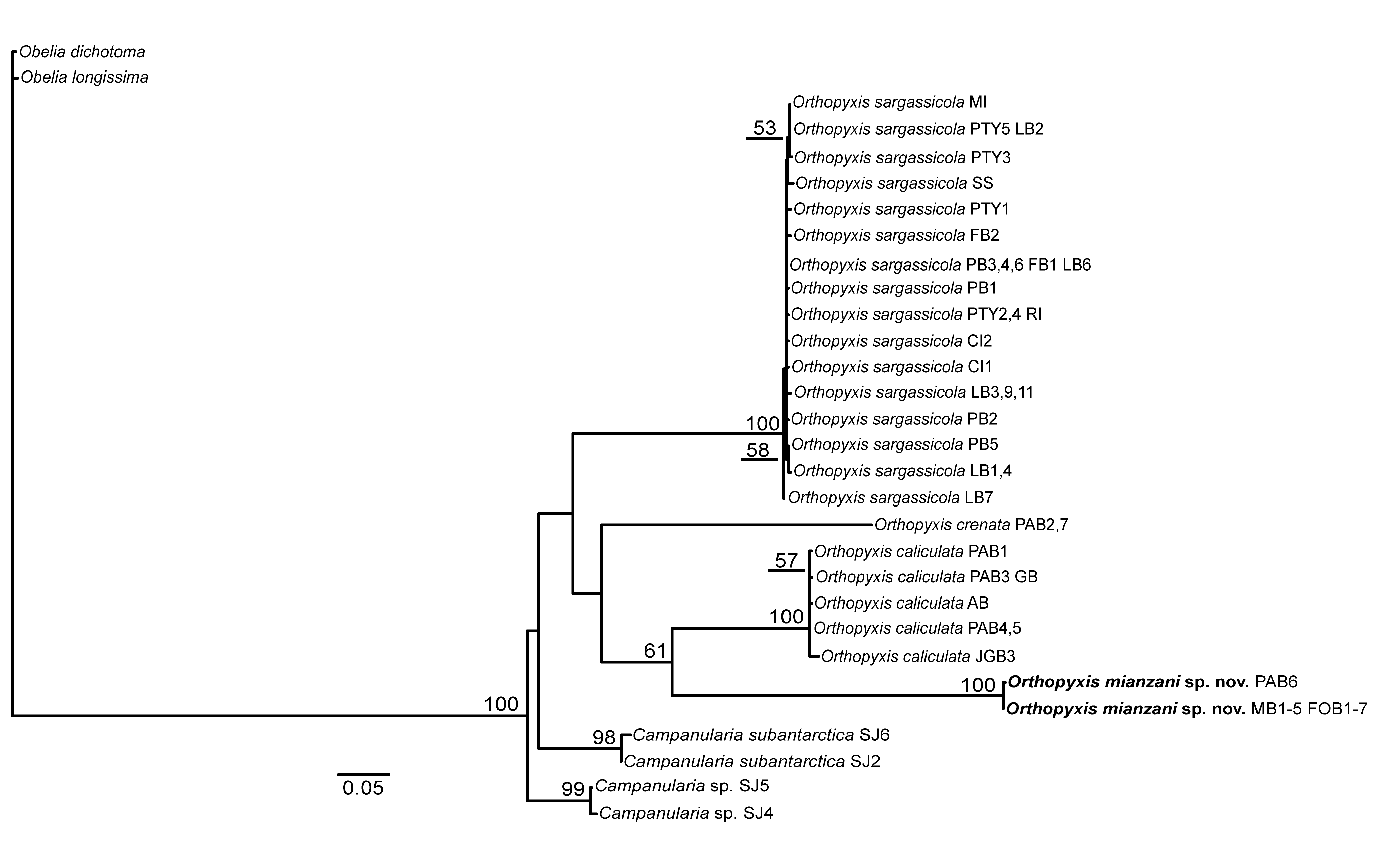

Supplement: S2 Fig — Bootstrap values are shown for each node. Nodes without numbers indicate support below 50. (TIF) [file pone.0117553.s002.tif]

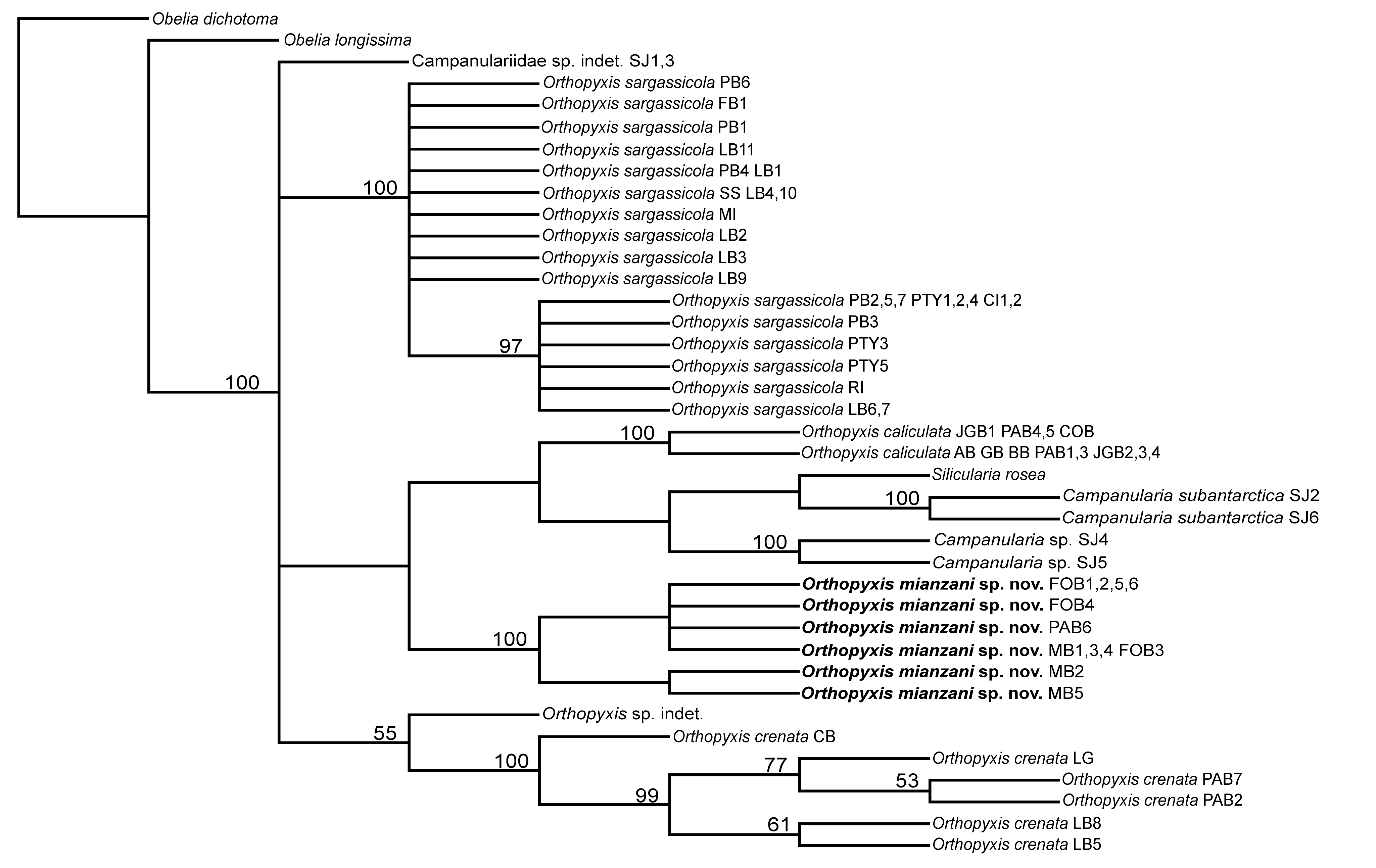

Supplement: S3 Fig — Bootstrap values are shown for each node. Nodes without numbers indicate support below 50. (TIF) [file pone.0117553.s003.tif]

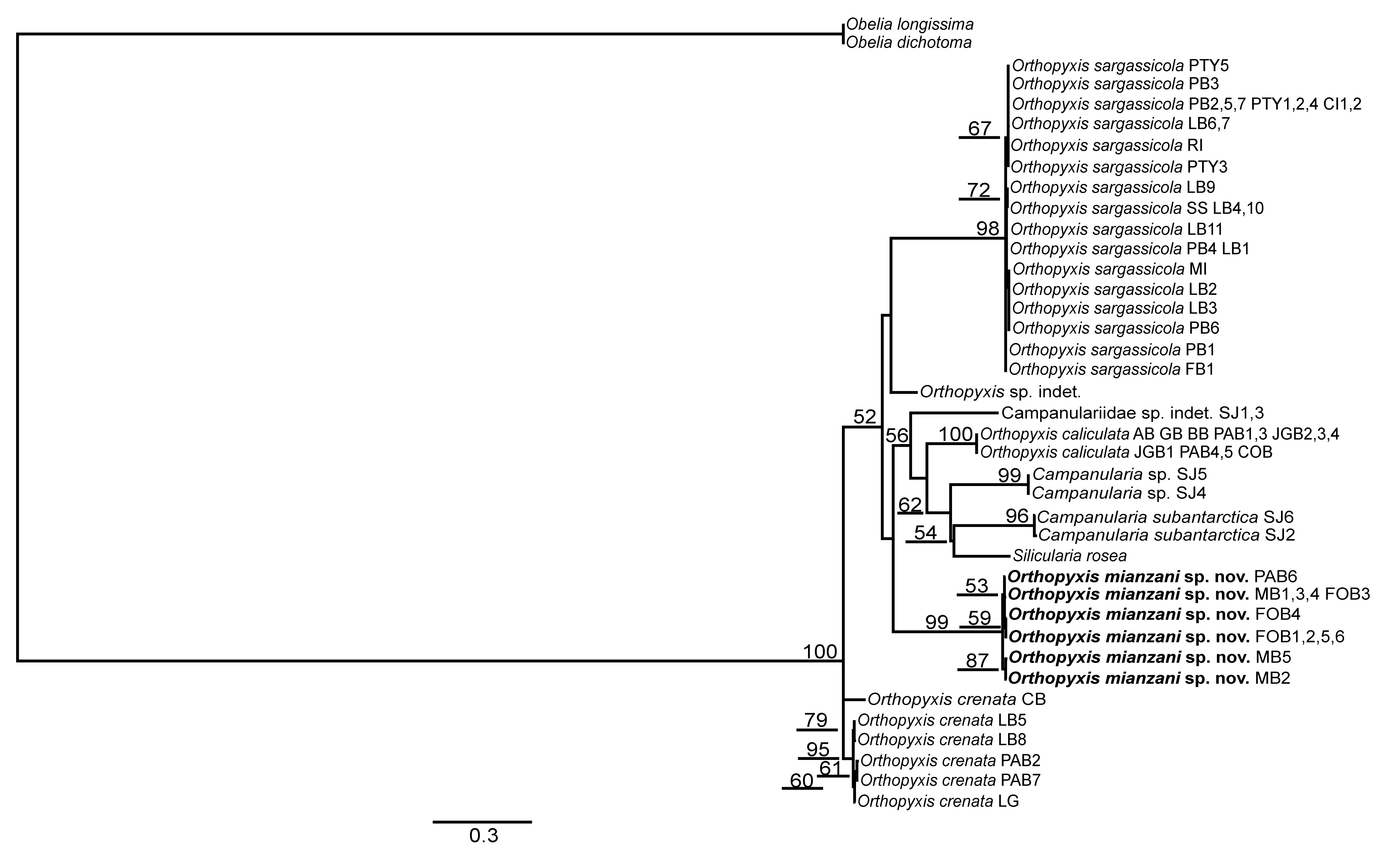

Supplement: S4 Fig — Bootstrap values are shown for each node. Nodes without numbers indicate support below 50. (TIF) [file pone.0117553.s004.tif]

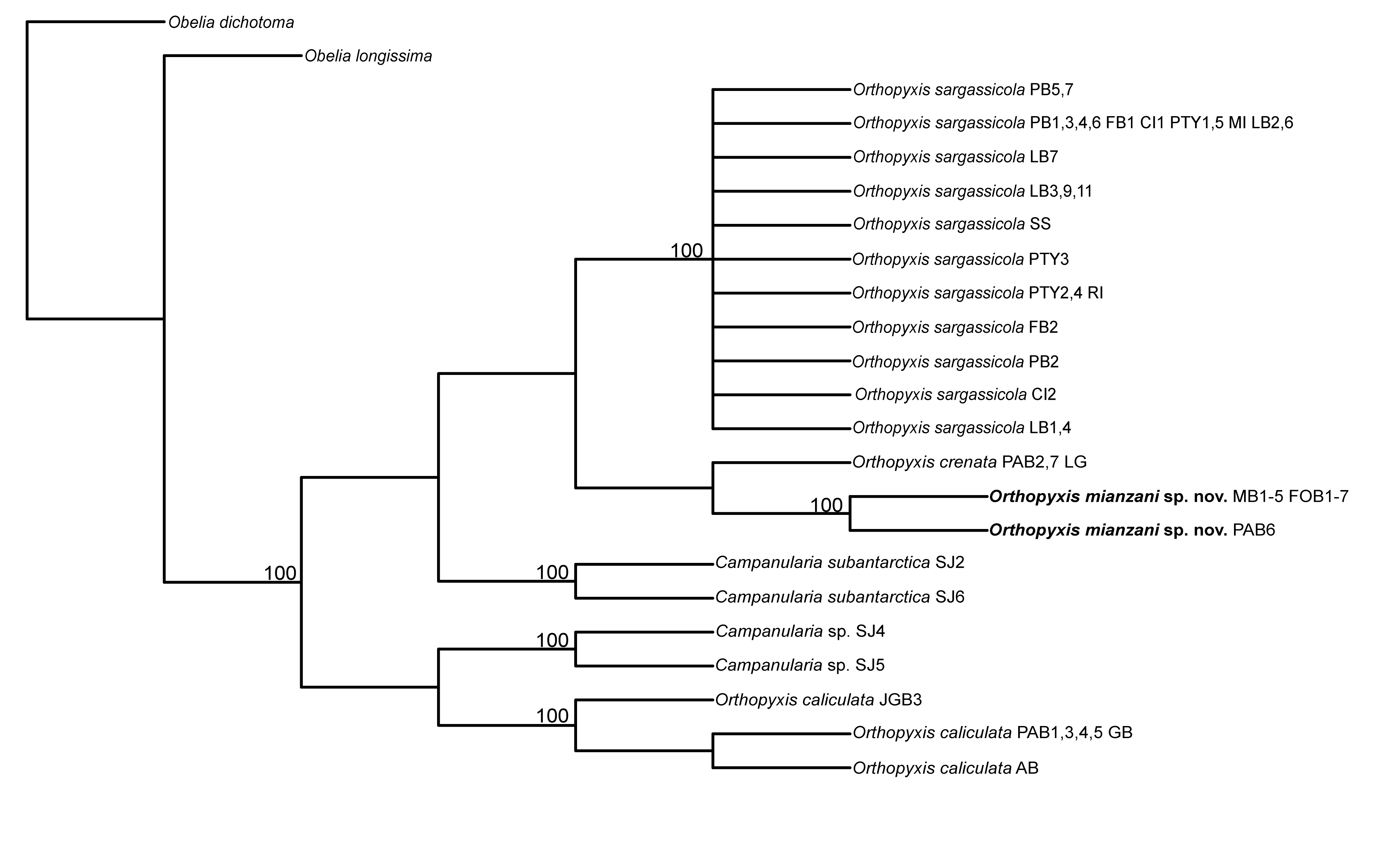

Supplement: S5 Fig — Bootstrap values are shown for each node. Nodes without numbers indicate support below 50. (TIF) [file pone.0117553.s005.tif]

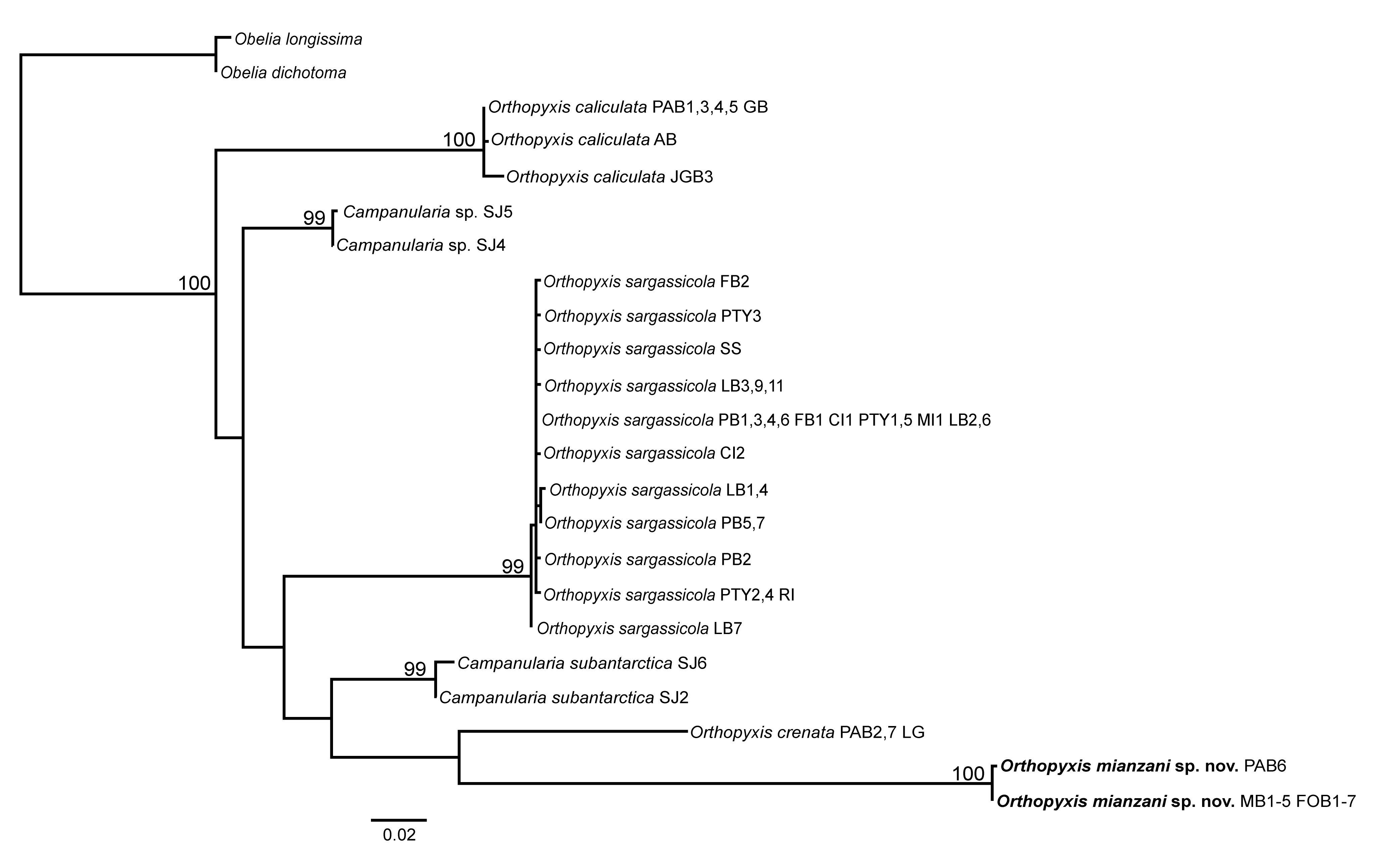

Supplement: S6 Fig — Bootstrap values are shown for each node. Nodes without numbers indicate support below 50. (TIF) [file pone.0117553.s006.tif]

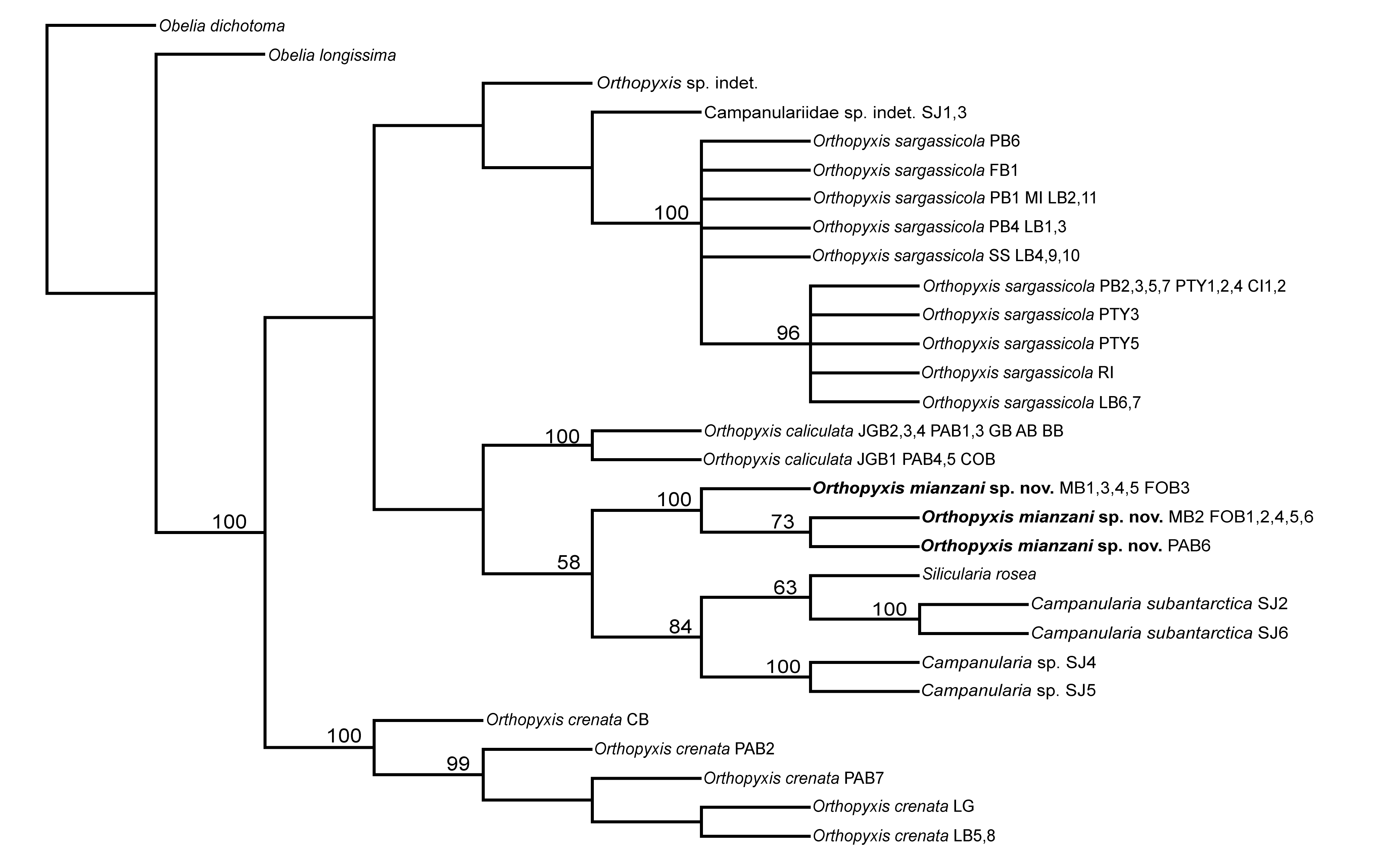

Supplement: S7 Fig — Bootstrap values are shown for each node. Nodes without numbers indicate support below 50. (TIF) [file pone.0117553.s007.tif]

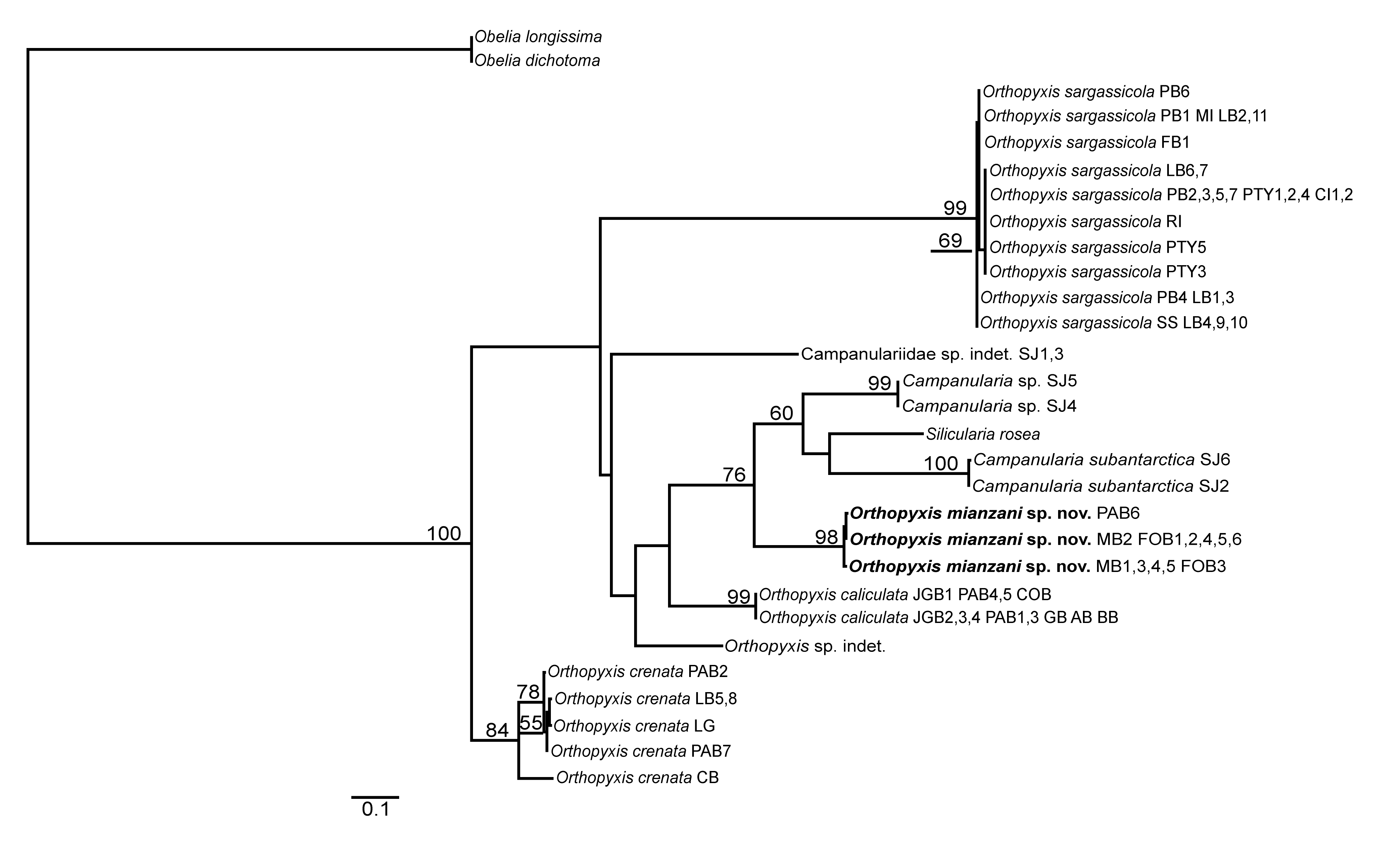

Supplement: S8 Fig — Bootstrap values are shown for each node. Nodes without numbers indicate support below 50. (TIF) [file pone.0117553.s008.tif]

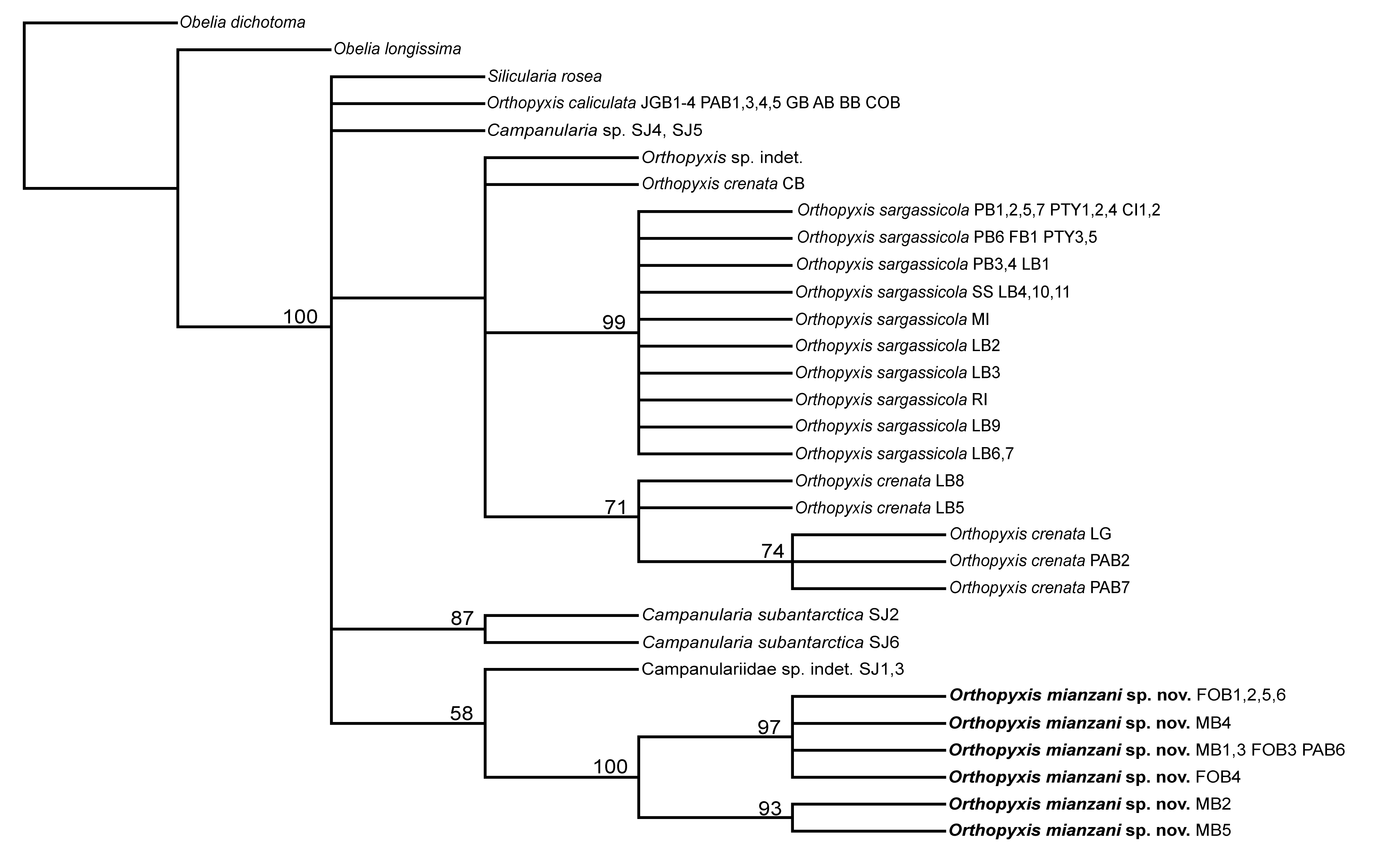

Supplement: S9 Fig — Bootstrap values are shown for each node. Nodes without numbers indicate support below 50. (TIF) [file pone.0117553.s009.tif]

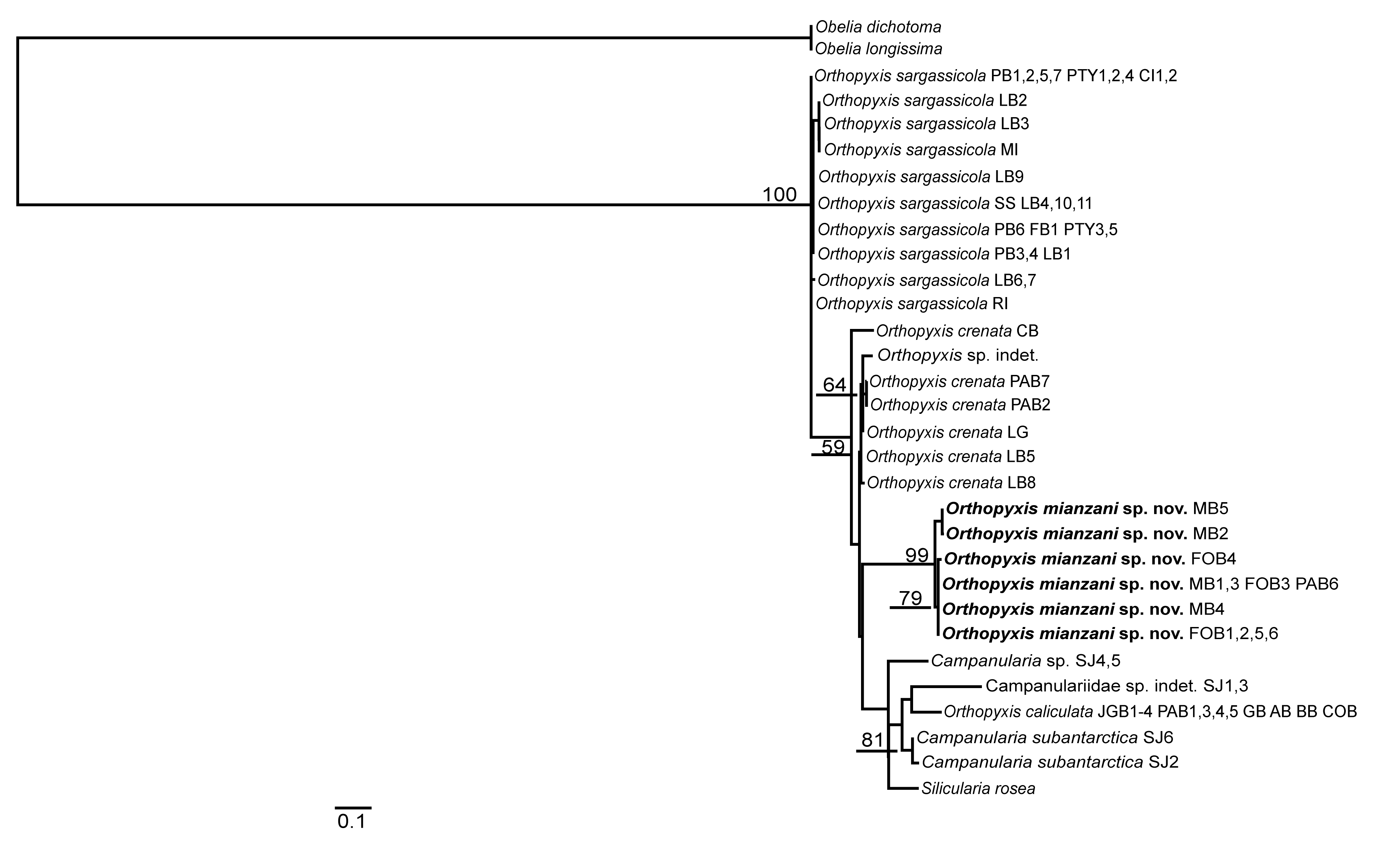

Supplement: S10 Fig — Bootstrap values are shown for each node. Nodes without numbers indicate support below 50. (TIF) [file pone.0117553.s010.tif]
