# Supplementary material for: Reassessment of Morphological Diagnostic Characters and Species Boundaries Requires Taxonomical Changes for the Genus Orthopyxis L. Agassiz, 1862 (Campanulariidae, Hydrozoa) and Some Related Campanulariids
Source: PLoS One. 2015 Feb 27;10(2):e0117553. doi: 10.1371/journal.pone.0117553 (PMC4344204; doi:10.1371/journal.pone.0117553)
Supplement: S1 Table — (DOCX) [file pone.0117553.s011.docx]

**Table S1. Morphological measures included in the Principal Component Analysis.**

| (1) Total length of the trophosome; |
| --- |
| (2) Diameter of hydrorhiza; |
| (3) Maximum perisarc thickness of hydrorhiza; |
| (4) Minimum perisarc thickness of hydrorhiza; |
| (5) Maximum perisarc thickness of hydrotheca at margin; |
| (6) Minimum perisarc thickness of hydrotheca at margin; |
| (7) Maximum perisarc thickness of hydrotheca at medial portion; |
| (8) Minimum perisarc thickness of hydrotheca at medial portion; |
| (9) Maximum perisarc thickness of hydrotheca at base; |
| (10) Minimum perisarc thickness of hydrotheca at base; |
| (11) Maximum perisarc thickness of pedicels at distal portion; |
| (12) Minimum perisarc thickness of pedicels at distal portion; |
| (13) Maximum perisarc thickness of pedicels at medial portion; |
| (14) Minimum perisarc thickness of pedicels at medial portion; |
| (15) Maximum perisarc thickness of pedicels at base; |
| (16) Minimum perisarc thickness of pedicels at base; |
| (17) Maximum perisarc thickness of subhydrothecal spherule; |
| (18) Minimum perisarc thickness of subhydrothecal spherule; |
| (19) Maximum diameter of subhydrothecal spherule; |
| (20) Minimum diameter of subhydrothecal spherule; |
| (21) Length of subhydrothecal spherule; |
| (22) Length of pedicel; |
| (23) Maximum diameter of pedicel at distal portion; |
| (24) Minimum diameter of pedicel at distal portion; |
| (25) Maximum diameter of pedicel at medial portion; |
| (26) Mininum diameter of pedicel at medial portion; |
| (27) Maximum diameter of pedicel at base; |
| (28) Minimum diameter of pedicel at base; |
| (29) Maximum number of sinuosities in the pedicel; |
| (30) Maximum number of constrictions in the perisarc of the pedicels; |
| (31) Length of hydrotheca; |
| (32) Maximum diameter of hydrotheca at margin; |
| (33) Minimum diameter of hydrotheca at margin; |
| (34) Maximum diameter of hydrotheca at medial portion; |
| (35) Minimum diameter of hdrotheca at medial portion; |
| (36) Maximum diameter of hydrotheca at base; |
| (37) Minimum diameter of hydrotheca at base. |
